# Fluorescent Polymers via Coordination of *bis*-Terpyridine Ligands with Transition Metals and Their pH Response Properties

**DOI:** 10.3390/polym17010087

**Published:** 2024-12-31

**Authors:** Tao Zhang, Fengxue Liu, Yongxin Liu, Kaixiu Li, Zhengguang Li, Yaqin Li, Fan Fu, Mingliang Liu, Yiming Li, Die Liu, Pingshan Wang

**Affiliations:** Department of Organic and Polymer Chemistry, College of Chemistry and Chemical Engineering, Central South University, Changsha 410083, China; 15273147569@163.com (T.Z.); liufengxue1065@163.com (F.L.); 19313057205@163.com (Y.L.); m18208989259@163.com (K.L.); chemicallee@163.com (Z.L.); 18373787608@163.com (Y.L.); 13875231276@163.com (F.F.); 18890020175@163.com (M.L.); chemyl@csu.edu.cn (Y.L.); chemwps@csu.edu.cn (P.W.)

**Keywords:** terpyridine, coordination polymer, transition metal complex, stimulus-response

## Abstract

Stimulus-responsive luminescent materials are pivotal in the field of sensing. Fluorescent transition metal complexes with a charge transfer excited state, especially terpyridine-coordinated polymers, are of particular interest due to their tunable emission. In this paper, a novel bis-terpyridine ligand was synthesized and assembled into a coordination polymer, which showed intense visible light absorption and fluorescence emission in the solid state that could be regulated by an acidic or basic pH. After being protonated by acid, the fluorescence of the polymer **P2** was quenched. The emission of the polymer split from 635 nm to two peaks of 674 and 440 nm, and then stabilized at 728 nm for 7 days, which showed a significant red-shift and good protonation stability. The fluorescence emission wavelength of the protonated polymers recovered after alkalization, and the fluorescence intensity of the polymer was greatly improved after alkalization, showing interesting acid–base-response luminescence characteristics. The sensitive response of the synthesized coordination polymers to acids and bases will contribute to expanding the application of linear coordination polymers in sensing and other fields.

## 1. Introduction

Photoluminescent materials are a class of substances that can emit visible light under excitation of an external light source [1]. They are widely used in testing devices, fluorescent labeling, display technology and biomedicine fields [2,3]. There are many kinds of photoluminescent materials, such as inorganic oxides, quantum dots, organic molecules and polymers [4]. Among these, transition metal complexes [5,6] play an important role in the field of photoluminescence due to their unique advantages, including high emission efficiency and environmental responsiveness [7], which makes them widely used in chemical sensing [8], luminescent devices [9], biomedicine [10,11] and other fields.

Terpyridine coordination polymers are a subclass of conjugated complexes in which multi-terpyridine ligands coordinate with metal ion coordinates to form polymer networks [12,13]. These polymers are known for their enhanced fluorescence properties, such as increased quantum yields, light stability, tunable emission wavelengths and responsive emission intensity, thanks to the rigidity and electronic communication conferred by coordination bonds [14]. And a wide range of applications have been developed, such as bioimaging as non-invasive probes, environmental analysis and detection of various analytes, including metal ions, anions and small molecules [15,16,17], and photo/electrocatalysis materials [18,19]. Current research areas mainly aim at exploring new terpyridine units to construct polymer scaffolders, and further study their coordination chemistry with different metal ions to afford functional materials [20,21,22]. There is growing interest in the design of polymers that can self-assemble into well-defined nanostructures with customized properties, as demonstrated by the pioneering work of various research groups around the world [23,24,25,26]. Among them, the *bis*-terpyridine coordination fluorescent polymer shows excellent photoelectric properties [27,28]. These structures exhibit unique properties at the nanoscale, providing new research directions for supramolecular chemistry and materials science. This kind of polymer adjusts the luminous color of the complex through the modification of the ligand, covering the range from purple to orange-red, and showing a dependence on the polarity of the solvent [29,30,31]. Moreover, this kind of ligand adjusts its polymerization degree by adjusting the reaction conditions of the coordination and shows different fluorescence and physical properties, which further broadens the application. In recent years, researchers have made progress in the synthesis of binuclear terpyridine ruthenium (II) complexes and their spectral and electrochemical properties. At the same time, the *bis*-terpyridine fluorescent coordination polymers are responsive to external stimuli such as pH, temperature, pressure, etc. [32,33], which creates potential value in applications in the field of smart materials and sensors.

Acid–base-responsive terpyridine fluorescent coordination polymers provide an important platform for pH-sensitive applications, in which variations in the emission intensity and lifetime can be observed. The acid–base response of a fluorescent coordination polymer usually results from the sensitivity of its ligand or metal center to changes in pH. With the change in the pH value, the protonation of ligands results in the ligand dissociation of polymers, which may affect the electronic structure and fluorescence properties of the polymer, resulting in changes in fluorescence intensity, wavelength or lifetime [32,34]. In the field of biosensing, fluorescent coordination polymers can be used as pH indicators to monitor tiny pH changes within cells, which is important for understanding cellular processes and disease diagnosis [35,36,37,38,39]. In addition, drug carriers of polymers display release behavior at specific pH values, which can be monitored in real-time via fluorescence signals [40]. For example, Yuan et al. designed a new pH-dependent fluorescence probe, which exhibited high sensitivity in an acidic environment with a working pH range of 7.2 to 2.5, especially having a good linear response to pH changes in the range of 2.5 to 4.3, and showed excellent pH probe performance [35]. In addition, terpyridine coordination polymers have extensive potential applications in biomedical imaging and environmental monitoring fields [41]. For example, terpyridine coordination polymers were successfully applied in second-harmonic generation bio-imaging within living cells and can be used as fluorescent probes for detecting heavy metal ions in aqueous solutions. Although fluorescence coordination polymers have made some progress in acid–base responsiveness, stimulus-responsive terpyridine coordination polymers with long emission wavelengths, fast response speeds and long-term stability are always desirable.

Here, we designed and synthesized two novel conjugated *bis*-terpyridine ligands **L1**/**L2**, which were further complexed with Zn^2+^ giving two linear fluorescent coordination polymers **P1**/**P2**. The UV-vis absorption spectra and fluorescence spectra of the two polymers **P2** and **P1** were analyzed. The fluorescence quantum yields of the two polymers were 9.56% and 9.43%, respectively. And their structure of linear polymers was well characterized by NMR, SEM, TEM and spectroscopic titrations. We focused on the response properties of **P2**, such as the acid–base response, and after being protonated, the fluorescence of **P2** was quenched. The emission of the polymer split from 635 nm to two peaks of 674 and 440 nm, and then stabilized at 728 nm for 7 days, which showed a significant red-shift and good protonation stability. Compared with the previous terpyridine coordination polymers, the ones in this paper have the advantages of longer wavelength emission, more stable protonation and visible acid–base responses [32,35].

## 2. Materials and Methods

**Scheme 1:**
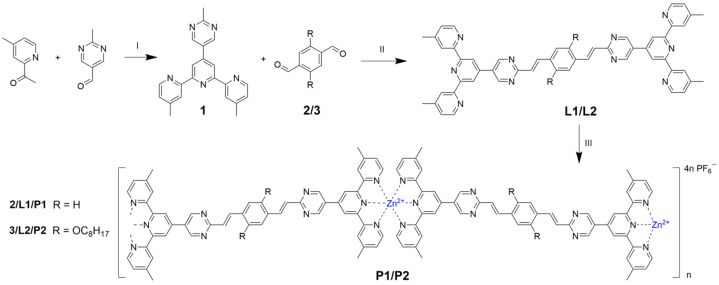
Synthesis route of the terpyridine ligands **L1**/**L2** and coordination polymers **P1**/**P2** (I: a. CH_3_CH_2_OH, NaOH; b. NH_3_·H_2_O, refluxed; II: KOH, THF, CH_3_OH, Ar, refluxed; III: Zn(NO_3_)_2_·6H_2_O, CHCl_3_/CH_3_OH).

Chemicals were purchased from Bide Pharmatech Co. Ltd., Sinopharm Group Co. Ltd., Adamas, Greagent, and used without further purification (Table S2). Thin layer chromatography (TLC) was conducted on flexible sheets (Baker-flex) precoated with Al_2_O_3_ (IB-F) or SiO_2_ (IB2-F). The synthesis of the monomer and the hydrogel with different compositions is described in Scheme 1. Column chromatography was conducted using basic Al_2_O_3_ Brockman Activity I (60–325 mesh) or SiO_2_ (60–200 mesh) from Fisher Scientific. NMR spectra were recorded on a Bruker NMR 400 or 500 MHz spectrometer (made in Bruker, Ettlingen, Germany), using CDCl_3_ for ligands and DMSO-d_6_ for metal coordination polymers with chemical shifts reported as ppm (TMS as internal standard). FT-IR was measured using a Bruker Vertex 70 Fourier transform spectrophotometer (made in Bruker, Ettlingen, Germany). Liquid fluorescence and solid fluorescence measurements were performed using the Hitachi F-4600 fluorescence spectrophotometer and Edinburgh FLS1000, respectively (made in the Edinburgh Instruments, Livingston, UK). The morphology of the hydrogels was determined using a scanning electron microscope (SEM, JEOL JSM-7500F) (made in JEOL Ltd., Tokyo, Japan). Before imaging, the samples were sputtered with Au. The TEM images of the samples were taken with a JEOL 2010 Transmission Electron Microscope (made in JEOL Ltd., Tokyo, Japan). The samples were dissolved in CH_3_CN at a concentration of ~10^−6^ M. The solutions were dropped cast onto a carbon-coated Cu grid (300–400 mesh), and the extra solution was blotted by filter paper to avoid aggregation.

### 2.1. Synthesis of Compound ***1***

Synthesis of compound **1**: 4-methyl-2-acetyl pyridine (2.65 g, 19.6 mmol), 2-methyl-5-pyrimidine formaldehyde (1 g, 8.2 mmol), 50 mL ethanol, 1.3 g NaOH were added into a round-bottomed flask. The system was stirred overnight, and then 50 mL of ammonia water was added. After refluxing for one day, the system was filtered after cooling. The filter residue was poured into a round-bottom flask, and 50 mL of isopropyl alcohol was added to reflux for 5 h. After cooling, the system was filtered, and the filter residue was dried overnight in a vacuum drying oven to obtain a light-yellow solid powder: 502 mg (yield: 10%). ^1^H NMR (400 MHz, CDCl_3_) δ: 9.12 (s, 1H, py-H^a^), 8.68 (s, 2H, Tpy-H^3′,5′^), 8.59 (d, J = 4.9 Hz, 2H, Tpy-H^6,6′′^), 8.47 (s, 2H, Tpy-H^3,3′′^), 7.21 (d, J = 4.9 Hz, 2H, Tpy-H^5,5′′^), 2.84 (s, 3H, py-CH_3_), 2.53 (s, 6H, Tpy-CH_3_). ^13^C NMR (101 MHz, CDCl_3_) δ: 168.56, 156.73, 155.46, 155.31, 149.09, 148.27, 144.00, 128.98, 125.21, 122.21, 118.47, 25.92, 21.41. ESI-MS (*m*/*z*): calcd: 354.1718; found: 354.2431 [M+H]^+^.

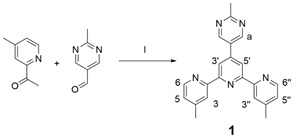


### 2.2. Synthesis of Ligand ***L1***

Synthesis of ligand **L1**: Compound **1** (500 mg, 1.16 mmol), terephthalaldehyde (65 mg, 0.486 mmol), KOH (200 mg), THF (40 mL) and CH_3_OH (20 mL) were added into a flask, and the system was degassed for 10 min and refluxed under Ar for 2 days. After cooling, the solvent was removed in vacuo to give a residue that was dissolved in CHCl_3_ and washed with CHCl_3_. The filter residue was dried overnight in a vacuum drying oven to obtain a green solid powder: 50 mg (yield: 5.4%). ^1^H NMR (400 MHz, CDCl_3_) δ: 9.23 (s, 4H, py-H^a^),8.74 (s, 4H, Tpy-H^3′,5′^), 8.61 (d, J = 5.0 Hz, 4H, Tpy-H^6,6′′^), 8.49 (s, 4H, Tpy-H^3,3′′^), 8.14–8.10, 7.42–7.38 (s, 4H, alkene-H^c,d^), 7.73 (s, 4H, Ph-H^b^), 7.22 (d, J = 5.0 Hz, 4H, Tpy-H^5,5′′^), 2.55 (s, 12H, Tpy-CH_3_). ESI-MS (*m*/*z*): calcd: 805.3516; found: 805.6388 [M + H]^+^.

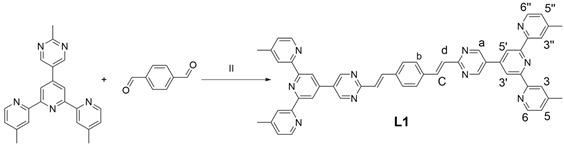


### 2.3. Synthesis of Ligand ***L2***

Synthesis of ligand **L2**: Compound **1** (500 mg, 1.16 mmol), 2,5-bis(octyloxy)terephthalaldehyde (190 mg, 0.486 mmol), KOH (200 mg), THF (40 mL) and CH_3_OH (20 mL) were added into a flask and the system was degassed for 10 min and refluxed under Ar for 2 days. After cooling, the solvent was removed in vacuo to give a residue that was dissolved in CHCl_3_, and then the solution was stirred for 5 h after adding CH_3_OH. The system was filtered, and the filter residue was dried overnight in a vacuum drying oven to obtain a yellow solid powder: 532 mg (yield: 43%). ^1^H NMR (400 MHz, CDCl_3_) δ: 9.22 (s, 4H, py-H^a^), 8.75 (s, 4H, Tpy-H^3′,5′^), 8.61 (d, J = 4.8 Hz, 4H, Tpy-H^6,6′′^), 8.48 (s, 4H, Tpy-H^3,3′′^), 8.44 (s, 2H, Ph-H^b^), 7.43–7.28 (d, J = 16.1 Hz, 4H, alkene-H^c,d^), 7.22 (d, J = 4.4 Hz, 4H, Tpy-H^5,5′′^), 4.10 (t, J = 6.5 Hz, 4H, OCH_2_), 2.54 (s, 12H, Tpy-CH_3_). ^13^C NMR (101 MHz, CDCl_3_) δ: 165.45, 156.70, 155.42, 155.34, 151.92, 149.06, 148.25, 144.15, 133.87, 128.58, 127.84, 127.21, 125.16, 122.20, 118.26, 111.56, 69.38, 31.86, 29.41, 29.36, 29.32, 26.15, 22.71, 21.41, 14.14. ESI-MS (*m*/*z*): calcd: 1061.5918; found: 1061.9525 [M + H]^+^.

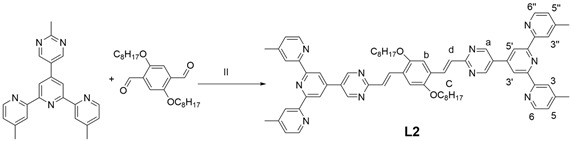


### 2.4. Synthesis of Ligand ***P1***

Synthesis of **P1**: ligand **L1** (50 mg, 0.062 mmol) and ZnNO_3_·6H_2_O (18.47 mg, 0.062 mmol) were added to CHCl_3_:CH_3_OH = 1:1 (100 mL). The mixture was refluxed for 16 h. Then excess NH_4_PF_6_ was added, generating a red precipitate which was washed with MeOH to give the desired product (65 mg, yield: 90.22%). ^1^H NMR (400 MHz, CD_3_SOCD_3_) δ: 9.73 (s, 4H, py-H^a^),9.31 (s, 4H, Tpy-H^3′,5′′^), 8.87 (s, 4H, Tpy-H^3,3′^), 8.78 (s, 4H, Tpy-H^c,d^), 7.80 (s, 4H, Tpy-H^5,5′′^), 7.27 (s, 4H, Ph-H^b^),6.70 (s, 4H, Tpy-H ^6,6′′^).

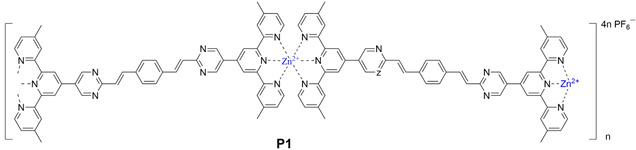


### 2.5. Synthesis of Ligand ***P2***

Synthesis of **P2**: ligand **L2** (50 mg, 0.047 mmol) and ZnNO_3_·6H_2_O (14 mg, 0.047 mmol) were added to CHCl_3_:CH_3_OH = 1:1 (100 mL). The mixture was refluxed for 16 h. Then excess NH_4_PF_6_ was added, generating a red precipitate which was washed with MeOH to give the desired product (60 mg, yield: 90%). ^1^H NMR (400 MHz, CD_3_SOCD_3_) δ: 9.87 (s, 1H, py-H^a^), 9.55 (s, 4H, Tpy-H^3′,5′′^),9.00 (s, 4H, Tpy-H^3,3′^), 8.62 (s, 4H, Tpy-H^c,d^), 7.82 (s, 4H, Tpy-H^5,5′′^), 7.68 (s, 2H, Ph-H^b^), 7.36 (s, 4H, Tpy-H ^6,6′′^), 4.28 (s, 4H, Ph-OCH_2_).

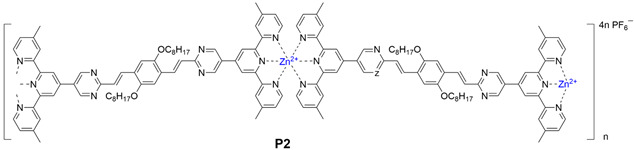


### 2.6. Synthesis of Ligand ***L3***

Synthesis of ligand **L3**: Compound **1** (200 mg, 0.466 mmol), 2,5-bis(octyloxy)benzaldehyde (337 mg, 0.931 mmol), KOH (78 mg), THF (40 mL) and CH_3_OH (20 mL) were added into a flask and the system was degassed for 10 min and refluxed under Ar for 2 days. After cooling, the solvent was removed in vacuo to give a residue that was dissolved in CHCl_3_, and then the solution was stirred for 5 h after adding CH_3_OH. The system was filtered and the filter residue was dried overnight in a vacuum drying oven to obtain a light green solid powder: 50 mg (yield:15%). ^1^H NMR (400 MHz, CDCl_3_) δ: 9.20 (s, 2H, py-H^a^), 8.74 (s, 2H, Tpy-H^3′,5′^), 8.60 (d, J = 5.1 Hz, 2H, Tpy-H^6,6′′^), 8.48 (s, 2H, Tpy-H^3,3′′^), 8.46 (s, 1H, Ph-H^f^), 8.42 (s, 1H, Ph-H^e^), 7.37 (d, J = 15.9 Hz, 2H, alkene-H^c,d^), 7.22 (d, J = 5.4 Hz, 2H, Tpy-H^5,5′′^), 6.87 (s, 1H, Ph-H^b^), 4.02 (t, J = 6.6 Hz, 2H, OCH_2_), 3.96 (t, J = 6.6 Hz, 2H, OCH_2_), 2.54 (s, 6H, Tpy-CH_3_). ^13^C NMR (101 MHz, CDCl_3_) δ: 171.25, 156.57, 156.57, 154.35, 153.17, 152.74–152.55, 151.06, 149.10, 144.88–144.27, 130.14, 128.16, 127.57, 125.07, 122.16, 118.32, 115.37–114.83, 112.74, 111.86, 68.83, 68.63, 59.96, 48.65, 40.95, 31.90, 31.86, 29.51, 29.47, 29.38, 29.25, 26.16, 26.11, 22.67, 22.55, 21.39, 14.10, 14.09. ESI-MS (*m*/*z*): calcd: 698.4434; found: 698.5745 [M + H]^+^.

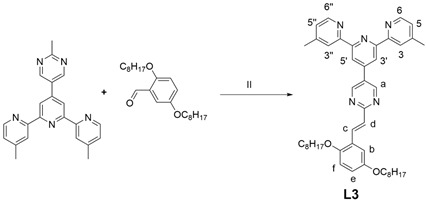


### 2.7. Synthesis of Complex ***[L3_2_Zn]***

Synthesis of complex **[L3_2_Zn]**: ligand **L3** (50 mg, 0.0717 mmol) and ZnNO_3_·6H_2_O (10.65 mg, 0.0359 mmol) were added to CHCl_3_:CH_3_OH = 1:1 (100 mL). The mixture was refluxed for 16 h. Then excess NH_4_PF_6_ was added, generating a red precipitate which was washed with MeOH to give the desired product (65 mg, yield: 90.22%). ^1^H NMR (400 MHz, CD_3_CN) δ: 10.12 (s, 4H, py-H^a^), 9.63 (s, 4H, Tpy-H^3′,5′^), 9.20 (s, 4H, Tpy-H^3,3′′^), 9.15 (d, J = 16.2 Hz, 2H, Ph-H^f^), 8.28 (t, J = 7.2 Hz, 4H, Tpy-H^6,6′′^), 8.13 (d, J = 16.1 Hz, 2H, Ph-H^e^), 7.97 (d, J = 2.8 Hz, 2H, Ph-H^b^), 7.87 (d, J = 5.2 Hz, 4H, alkene-H^5,5′′^), 7.68–7.57 (m, 4H, Tpy-H^c,d^), 4.72 (t, J = 6.6 Hz, 4H, OCH_2_), 4.65 (t, J = 6.5 Hz, 4H, OCH_2_), 3.12 (s, 12H, Tpy-CH_3_). ESI-MS (*m*/*z*): +2 (*m*/*z* = 730.5012) (calcd: *m*/*z* = 731.4086).

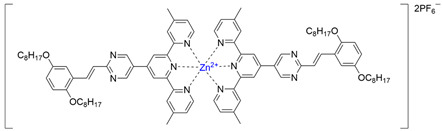


## 3. Results and Discussion

### 3.1. Synthesis and Characterization

**Figure 1:**
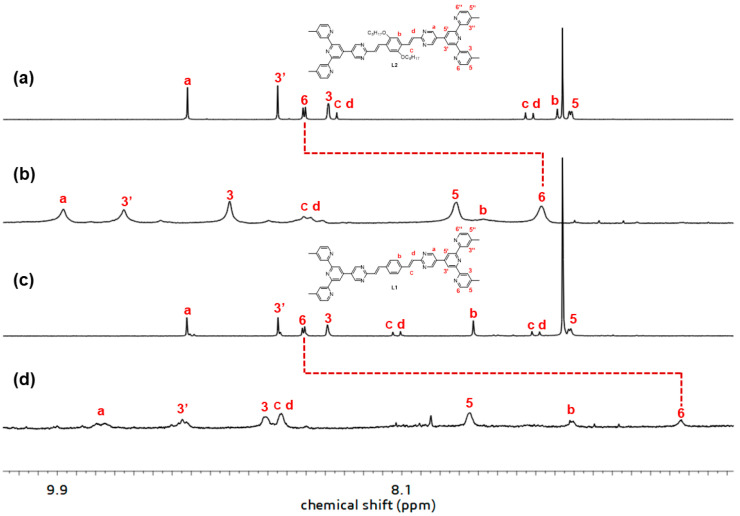
^1^H NMR spectra of (**a**) **L2** (CDCl_3_), (**b**) **P2** (DMSO-d_6_), (**c**) **L1** (CDCl_3_) and (**d**) **P1** (DMSO-d_6_).

The metallo-macrocycle **P1** and **P2** were self-assembled by directly mixing the ligand **L2, L1** respectively, and Zn(NO_3_)_2_ in a stoichiometric ratio of 1:1 in CH_3_CN/CH_3_OH (*v*/*v*, 1/1), with stirring at 75 °C for 8 h. Subsequently, a saturated CH_3_OH solution of NH_4_PF_6_ was added to counter anions exchange. Ligands **L2**, **L1** and coordination polymers **P2** and **P1** were characterized by NMR (^1^H), and the NMR signal peaks of **P2** were assigned with the aid of 2D COSY and 2D NOESY. The ^1^H NMR spectra of the coordination polymer **P2** (Figure 1a) showed that the peaks in the aromatic region were all very broad components, which were caused by the formation of the aromatic π pile and the mutual shielding of the aromatic ring. Similarly, even if less obvious, spectral signatures of the alkyl chain portion bound to the heterocyclic were detected in the aliphatic region, indicating that it was affected by ring current shielding and de-shielding phenomena, which are consistent with the nuclear magnetic characteristics of the polymer [42]. In the 2D COSY spectrum, there were two sets of clearly related signal peaks with an integral ratio of 1:1, which can be assigned to the H^5,5′′^, H^6,6′′^ protons of tpys. In addition, the ^13^C NMR signals of complex **L3_2_Zn** between 145 ppm and 157 ppm showed a significant shift in comparison with ones of ligand **L3** (Figure S16), and a band at 1400 cm^−1^ assigned to coordinated pyridines was observed in the FT-IR spectrum, which proved the successful coordination of the ligand **L3** with Zn^2+^ (Figure S25). In the 2D NOESY spectrum, protons at 9.55 ppm appeared to cross peaks with other tpy protons, which can be assigned to the H^3,3′′^ proton of tpys. In addition, in the non-aromatic region, only a characteristic peak of methylene oxide was observed at 4.28 ppm, indicating the uniqueness of the product and the high symmetry of the structure. The remaining localization was carefully confirmed by ^1^H NMR. The analysis of **P1** was similar to that of **P2**. All of these indicated that the targeted polymers were successfully synthesized.

### 3.2. Microstructures

**Figure 2:**
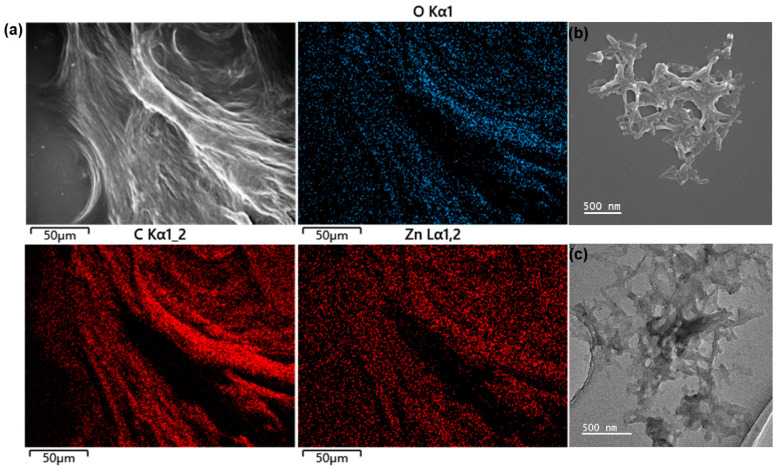
(**a**) SEM and mapping (EDS) images of **P2**; (**b**) SEM image of **P1**; (**c**)TEM image of **P1**.

To gain a deeper insight into the microstructure within the polymers, solid and solution samples were characterized using scanning electron microscopy (SEM) and transmission electron microscopy (TEM). The ligand **L2** was a rigid conjugated molecule, and the solid polymer **P2** formed from **L2** showed a long filament shape observed from SEM profiles (Figure 2a), which is according to our initial morphological prediction. In addition, the energy dispersive X-ray spectroscopy (EDS) mapping technique (Figure 2a and Figure S22) was performed on the **P2** solid, which mainly contained C and O elements, as well as N and Zn elements, which were consistent with the molecular composition of the polymer, and the target coordination polymer was further determined. In acetonitrile solution, due to the high solubility, the polymer P2 displayed spherical and wormlike morphology (Figure S24). **L1**, the precursor of polymer **P1**, lacked long chains and was less soluble in organic solvents than **L2**. **P1** had a slower solubility of polymerization and appeared as a compact rode-like structure (Figure 2b,c). Polymer **P2** could be evenly distributed into organic solvents, such as acetonitrile, and the molecular size was about 20–30 nm (Figure S23). These polymer molecules could be deposited to form fibrous morphology, which has a stable structure and uniform morphology in both solid and solution states. The ligand **L2** was a conjugated system formed by the connection of terpyridine and the benzene ring through C=C. After coordination polymerization, the polymers generated a clear π–π stacking and enhanced charge transfer, resulting in a redshift of fluorescence emission from the bright yellow emission of the ligands to the orange-red emission of the polymers (detailed discussion can be found below in Section 3.4 Optical Properties of Polymers). The fluorescence color change was consistent with the structural change, which was also evidence for the synthesis of the target polymer.

### 3.3. Job Plots and Ultraviolet-Visible Spectroscopic Titrations

**Figure 3:**
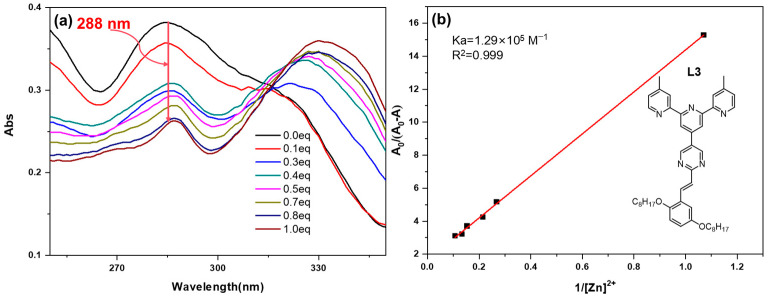
(**a**) Abs of mixture of **L3** (9.316 μM) and [Zn^2+^] (0.0–1.0 eq) stirred for 4 h. (**b**) Plot of (A_0_/(A − A_0_) as a function of 1/[Zn^2+^]. The apparent association constant Ka corresponding to the interaction between **L3** (9.316 μM) and [Zn^2+^] (0.0–1.0 eq) was determined using the Benesi–Hilderbrand equation A_0_/(A − A_0_) = (A_0_/(Amax − A_0_))((1/Ka)[Zn^2+^]^−1^ + 1).

To obtain further insights into the coordination between **L2** and Zn(NO_3_)_2_, the complexation between ligand **L3** and Zn(NO_3_)_2_ was studied using the Job method [43]. As shown in Figure 3a, 0.0–1.0 eq Zn(NO_3_)_2_ was added to a dilute solution of **L3**/CHCl_3_/CH_3_OH (1/1, *vol*/*vol*). After stirring for 4 h, the absorption peak at 288 nm was weakened significantly as the molar amount of Zn(NO_3_)_2_ increased. At the same time, the new absorption peak at 331 nm was significantly enhanced, which indicated that **L3** was gradually transformed into a new substance after the addition of Zn(NO_3_)_2_. The coordination of metal ion Zn^2+^ with ligand **L3** atom rendered an octahedral configuration containing two ligands and one Zn^2+^ [44]. The relationship between the absorbance value of **L3** at 288 nm and the concentration of Zn(NO_3_)_2_ was established by using the Benesi–Hildebrand equation to construct the titration curve (Figure 2b) [45,46], so that the complex constant Ka between **L3** and [Zn^2+^] was calculated as 1.29 × 10^5^ M^−1^ which was also generalized to the complexation between **L2** and [Zn^2+^]. After calculating the complexation constant from the ultraviolet-visible spectra titration experiment between **L3** and [Zn^2+^], we followed the formula DP ≈ (KaC)^1/2^ to calculate the degree of polymerization of **P2** [30,47,48]. According to the formula, the DP_cal_ was calculated to be 7.8 (C = 4.71 × 10^−4^ M), while according to the simulation, the length of the monomer was about 3.11 nm, and the size of the polymer molecule (DP = 8) was calculated to be 22.41 nm (Figure S25). In transmission electron microscope (TEM) images, individual molecules were 20–30 nm in size (Figure S24). The theoretical calculation was in agreement with the experimental results, which further showed that the synthesized coordination polymer was in agreement with the expectation.

### 3.4. Optical Properties of Polymers

**Figure 4:**
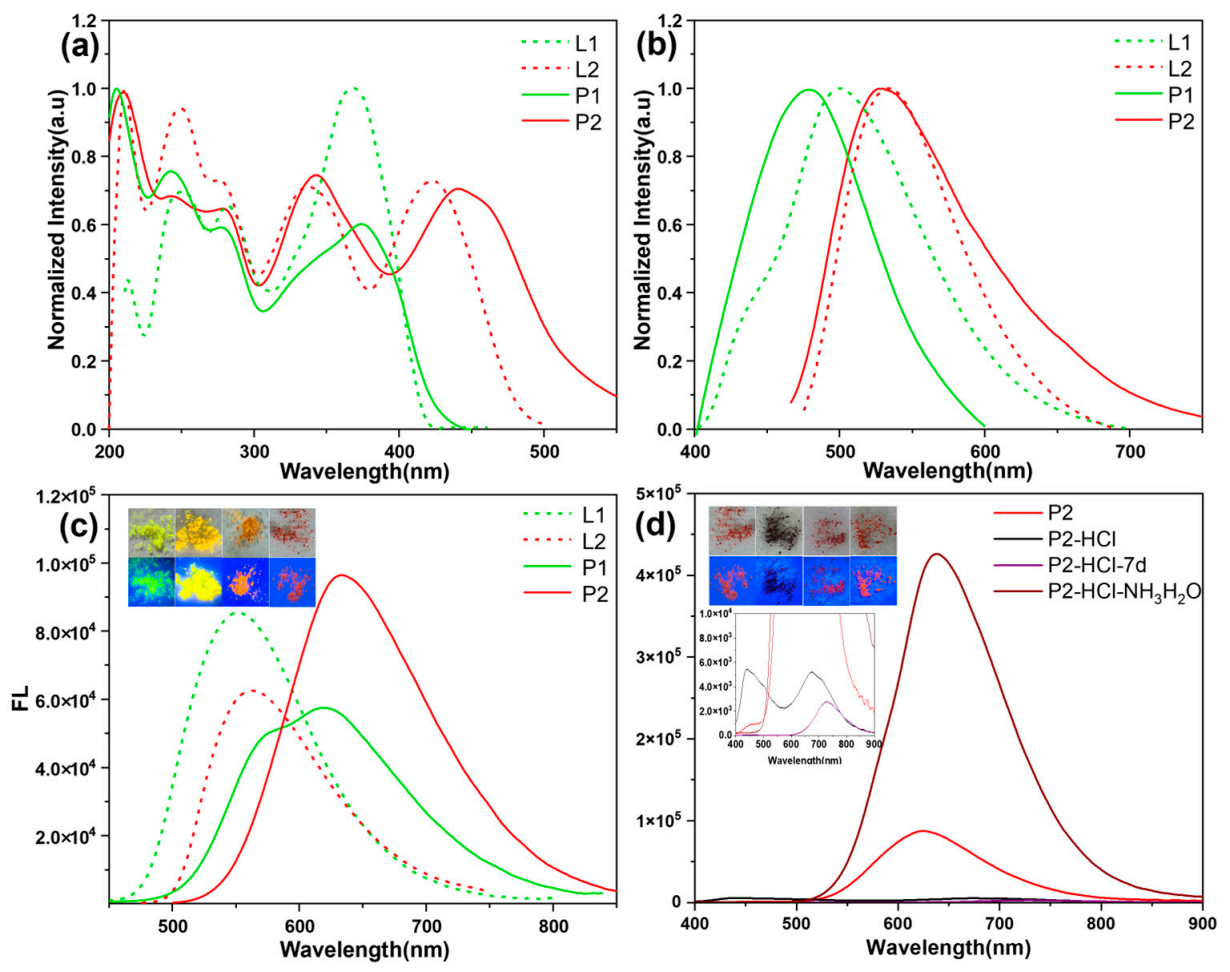
(**a**) UV-Vis and (**b**) Fluorescence spectrum of **L1**, **L2**, **P1**, **P2** (1 × 10^−5^ mol·L^−1^ in CH_3_CN), LOD and LOQ of **L1**, **L2**, **P1**, **P2** can be seen in Figure S29; (**c**) Fluorescence spectrum of solid **L1**, **L2**, **P1**, **P2**; (**d**) Acid–base response of **P2**, the Fluorescence spectrum of solid **P2**, **P2** fumigated with concentrated hydrochloric acid (30 s), fumigated **P2** after 7 days and fumigated **P2** fumed with ammonium hydroxide. Inset: Photos of ligands and polymers under sunlight (**top**) and ultraviolet light (**bottom**).

The emission peaks of **P2** and **P1** were 535 nm and 480 nm, respectively, while **P2** had three absorption peaks at 215, 350 and 450 nm, and **P1** had two absorption peaks at 265 and 330 nm (Figure 4a). Compared with the UV-Vis spectrum of **P1**, **P2** had an extra absorption peak in the visible region. This may be because **P2** had two more flexible long chains on the intermediate benzene ring, whose spatial volume and shape affect the planar configuration of the polymers, thus affecting the integrity of the conjugated system and resulting in the distortion of the molecular structure, reducing the conjugated length, and thus leading to the splitting of the absorption peaks. Compared with the absorption wavelength of **P1**, the absorption peak wavelength of **P2** was red-shifted, which may be due to the conjugation of the substituent with the large π bond, which increased the electron cloud density and increased the degree of electron delocalization in the molecule. This delocalization reduced the energy level difference of the electron transition, reducing the energy required for the electrons in the molecule to transition from the ground state to the excited state, causing the absorption peak to shift to a longer wavelength, and the emission wavelength followed the same principle. The side chain alkyl of **P2** monomer improved the solubility and provided a suitable steric hindrance to prevent the fluorescence quenching caused by excessive stacking of **P2** molecules. **P2** solid had a longer wavelength of fluorescence emission than its acetonitrile solution (Figure 4b,c), which is attributed to the stronger intermolecular interaction of the polymer in the solid. And π–π deposition increased the intermolecular conjugation, thereby reducing the energy required for electron transition, resulting in enhanced fluorescence of the solid, and a longer wavelength of fluorescence. This high-efficiency luminescence is thought to be related to the formation of intermolecular hybridized local and charge–transfer (HLCT) excited states, which induce the reduction in the π–π distance between molecules and enhance the intermolecular rigidity, thus the non-radiative rate is suppressed more effectively [49]. Compared with the complex, the steady-state solid fluorescence of the coordinated polymer was significantly red-shifted (Figure 4c), which is attributed to the coordination of the ligand with the metal ion. After coordination, the polymer had a separated charge center, resulting in a stronger built-in electric field between the anions, which promoted charge transfer and the exciton dissociation efficiency.

### 3.5. pH-Responsive Properties

**Figure 5:**
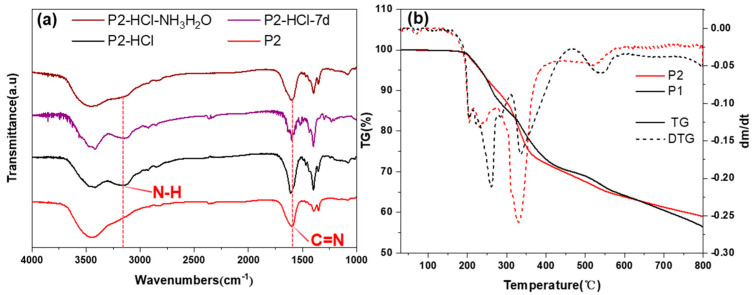
(**a**) Infrared spectrum of solid **P2**, **P2** fumigated with concentrated hydrochloric acid (30 s), fumigated **P2** after 7 days and fumigated **P2** fumed with ammonium hydroxide; (**b**) Thermogravimetric and differential thermogravimetric analysis diagram of **P1** and **P2** (the red line represents **P2**, the black line represents **P1**, the solid line represents thermogravimetric analysis, and the dashed line represents differential thermogravimetric analysis).

Compared to the original polymer **P2**, the FT-IR spectrum of **P2** after acidification in a concentrated hydrochloric acid atmosphere was a new peak at 3300–3000 cm^−1^, which is attributed to the N-H stretching vibration of the protonated pyridine ring (Figure 5a). In addition, the peak at 1587 cm^−1^ showed a 20 cm^−1^ shift after acidification, indicating that the C=N bond stretching vibration was significantly affected after acidification, which is attributed to the fact that acidification changed the dipole moment of the molecule, thus affecting the infrared activity of the vibration mode [50,51,52]. All these indicated that the N on the terpyridine ring bound to the proton after acidification, affecting the conjugation of the polymer. **P2** solid fluorescence had an emission wavelength of 635 nm, and after acidification, the emission peak was obviously quenched and red-shifted, which may be due to the excessive protonation of the terpyridine ring (Figure 4d). For 7 days after protonation, the slight red-shifted fluorescence emission wavelength of the polymer was in the near-infrared region, which is attributed to enhanced electron-withdrawing ability after protonation of the pyridine ring. Thus, enhanced charge transfer transition renders the energy required for the electron transition be reduced, resulting in the fluorescence emission wavelength moving in the direction of the longer wavelength. In addition, the charge density of the partially protonated polymer was not uniform around the terpyridine unit, which greatly enhanced its inherent ability to separate photogenerated charges, so that the polymer electrons were more easily excited and the fluorescence wavelength was longer. After acidification, the polymer **P2** maintained a protonated state for a long time, and maintained stability for more than one week without external interference, which is attributed to the strong ability of **P2** to bind protons, and the polymer fiber packing structure can adsorb protons well. The isolated polymer **P2** maintained a stable protonation state for a long time, and after 30 s in a weak alkali atmosphere, the fluorescence wavelength of **P2** solids returned to close to the pre-protonation, the N-H stretching vibration peak disappeared, and the C=N stretching vibration peak recovered in FT-IR. All these indicated that the protonated **P2** can quickly deprotonate after a little alkalization, and return to close to the original level. Moreover, the fluorescence emission intensity of the protonated polymer after alkalization was 4.9 times higher than that before unprotonation, showing surprising fluorescence properties. In addition, the protonation was a gradual process that was completed in a very short time (30 s) and stood for a long time in a closed environment (Figure S30), while the process of deprotonation was greatly accelerated in an alkaline environment (Figure 4d). The rapid transformation of the polymer **P2** under protonation and deprotonation, the great enhancement of its fluorescence properties, and its good thermal stability (Figure 5) have expanded the application of the polymers **P1** and **P2** in the fields of photocatalysis, conductive materials, biomarkers and so on.

## 4. Conclusions

The *bis*-terpyridine fluorescent coordination polymer developed in this study exhibited remarkable protonation stability and acid–base responsiveness, with its fluorescence properties changed significantly after protonation. Its fluorescence intensity was greatly enhanced while the fluorescence emission wavelength was easily recovered by alkalization. The polymer’s ability to remain protonated for a long time, combined with its rapid deprotonation and enhanced fluorescence intensity, underscores its potential in a variety of applications, including photocatalysis, conductive materials, and biomarkers. The results in this paper also indicated that the flexible side-chain groups in polymer **P2** have important effects on solubility, morphology, and luminescence performance, which provides an effective way to customize materials with specific properties. Such results were consistent with previous reports, in which the number, position and length of side-chain groups greatly affect the properties of polymer, such as the intermolecular interaction, crystal orientation and device performances [53,54,55,56]. Future research will concentrate on enhancing response speed, selectivity and stability, as well as exploring novel ligands and metal centers to create more complex supramolecular systems with diverse response properties. This study lays a solid foundation for the future development of smart photoluminescent materials for advanced technological applications.

## Data Availability

The data are contained within the article and the Supplementary Materials.

## Supplementary Materials

The following supporting information can be downloaded at: https://www.mdpi.com/article/10.3390/polym17010087/s1, Figure S1. ^1^H NMR spectrum of compound **1** in CDCl_3_. Figure S2. ^1^H NMR spectrum of compound **3** in CDCl_3_. Figure S3. ^1^H NMR spectrum of ligand **L2** in CDCl_3_. Figure S4. ^1^H NMR spectrum of ligand **L1** in CDCl_3_. Figure S5. ^1^H NMR spectrum of ligand **L3** in CDCl_3_. Figure S6. ^1^H NMR spectrum of polymer **P2** in DMSO-d_6_. Figure S7. ^1^H NMR spectrum of polymer **P1** in DMSO-d_6_. Figure S8. ^1^H NMR spectrum of complex [**L3_2_Zn**] in CD_3_CN. Figure S9. 2D COSY NMR spectrum of polymer **P2** in DMSO-d_6_. Figure S10. 2D COSY NMR spectrum of complex [**L3_2_Zn**] in CD_3_CN. Figure S11. 2D NOESY NMR spectrum of **P2** in DMSO-d_6_. Figure S12. 2D NOESY NMR spectrum of complex [**L3_2_Zn**] in CD_3_CN. Figure S13. ^13^C NMR spectrum of compound **1** in CDCl_3_. Figure S14. ^13^C NMR spectrum of ligand **L2** in CDCl_3_. Figure S15. ^13^C NMR spectrum of ligand **L3** in CDCl_3_. Figure S16. ^13^C NMR spectra of **L3** and **L3_2_Zn**. Figure S17. ESI-MS spectrum of compound **1**. Figure S18. ESI-MS spectrum of ligand **L1**. Figure S19. ESI-MS spectrum of ligand **L2**. Figure S20. ESI-MS spectrum of ligand **L3**. Figure S21. ESI-MS spectrum of complex [**L3_2_Zn**]. Figure S22. SEM and mapping images of solid **P2**. Figure S23. SEM and mapping images of solid **P1**. Figure S24. (a) SEM and (b) TEM images of **P2**; (c) SEM and (d) TEM images of **P1**. Figure S25. FT-IR spectrum of **L2**, **L3**, **P2** and **L3_2_Zn**. Figure S26. Fluorescence quantum yield of **P2**. Figure S27. Fluorescence quantum yield of **P1**. Figure S28. Photographs of **L1**, **L2**, **P1**, **P2**, **P2**-HCl, **P2**-HCl-7day, **P2**-HCl-NH_3_H_2_O under daylight and ultraviolet light. Figure S29. Fluorometric titration diagram of **L2**, **L1**, **P1**, **P2**. LOD(**L2**) = 3δ/k = 3 × 2.1317÷159.77 × 10^−5^M = 4.00 × 10^−7^ M, LOQ(**L2**) = 10δ/k = 1.22 × 10^−6^ M; LOD(**L1**) = 3δ/k = 6.8 × 10^−7^ M, LOQ(**L1**) = 10δ/k = 2.2 × 10^−6^ M; LOD(**P1**) = 3δ/k = 5.55 × 10^−7^ M, LOQ(**P1**) = 10δ/k = 1.85 × 10^−6^ M; LOD(**P2**) = 3δ/k = 2.8 × 10^−7^ M, LOQ(**P2**) = 10δ/k = 9.44 × 10^−7^ M. Figure S30. Time-dependent solid fluorescence spectra of polymer **P2** after protonation. Table S1. The parameters of geometry optimization by using Forcite Calculation of Material Studio. Figure S31. Geometry optimization molecule model and corresponding size of (a) complex [**L3_2_Zn**], (b) polymer **P1** (DP = 8) and (c) polymer **P2** (DP = 8). Table S2. The provider and purity of chemical reagents.
