# Supplementary material for: Fluorescent Polymers via Coordination of bis-Terpyridine Ligands with Transition Metals and Their pH Response Properties
Source: Polymers (Basel). 2024 Dec 31;17(1):87. doi: 10.3390/polym17010087 (PMC11723069; doi:10.3390/polym17010087)
Supplement: Supplementary file 1 [file polymers-17-00087-s001.zip › polymers-3353947-supplementary.pdf]

Fluorescent Polymers via Coordination of *bis*-Terpyridine Ligands with Transition Metals and Their pH Response Properties

S1 NMR **S1**

S2 ESI-MS spectra **S10**

S3 SEM and TEM images **S12**

S4 Optical property **S14**

S5 Molecular model **S17**

S1 NMR

^{1}H NMR spectra

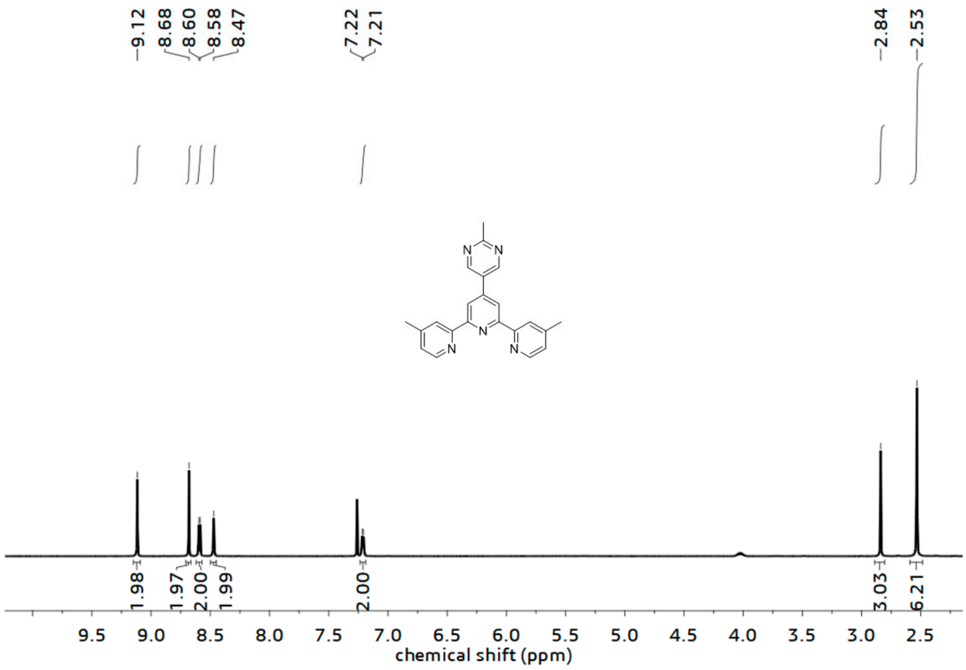

Figure S1. ^{1}H NMR spectrum of compound **1** in $CDCl_3$.

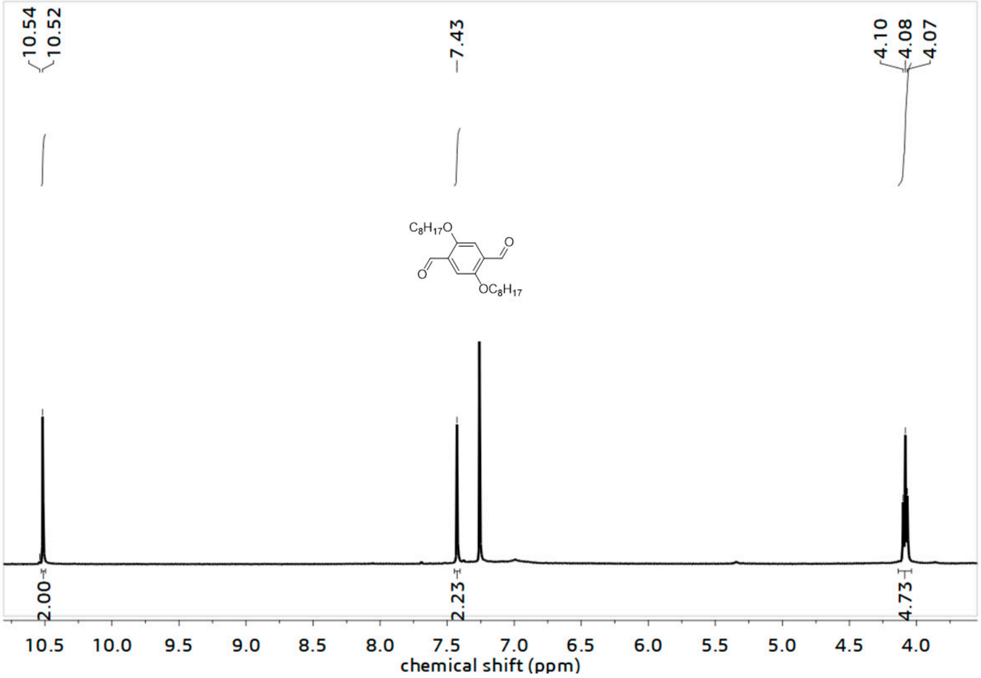

Figure S2. ^{1}H NMR spectrum of compound **3** in $CDCl_3$.

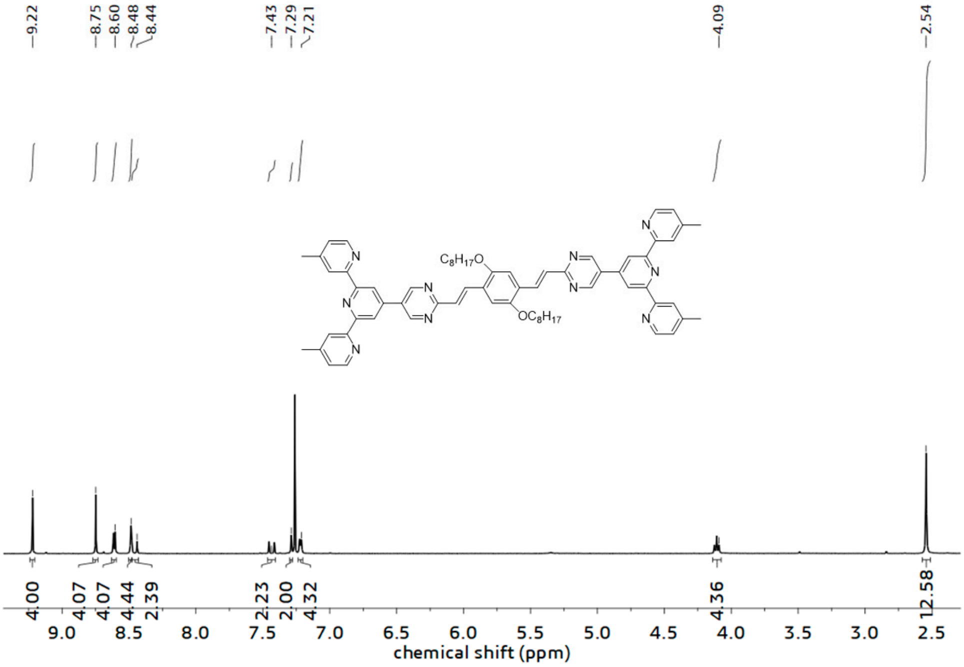

Figure S3. ^{1}H NMR spectrum of ligand **L2** in $CDCl_3$.

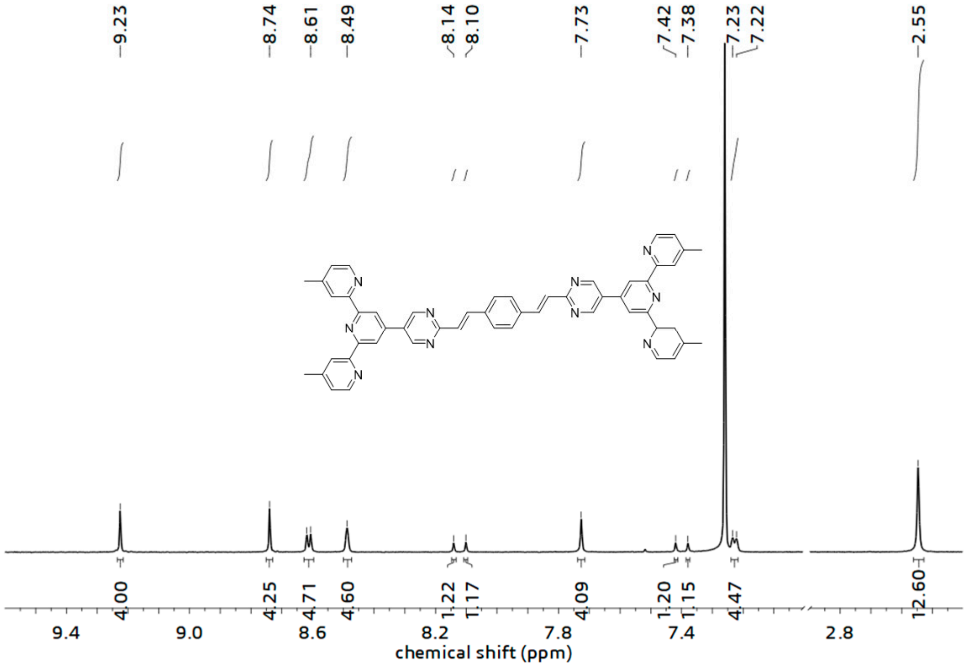

Figure S4. ^{1}H NMR spectrum of ligand **L1** in $CDCl_3$.

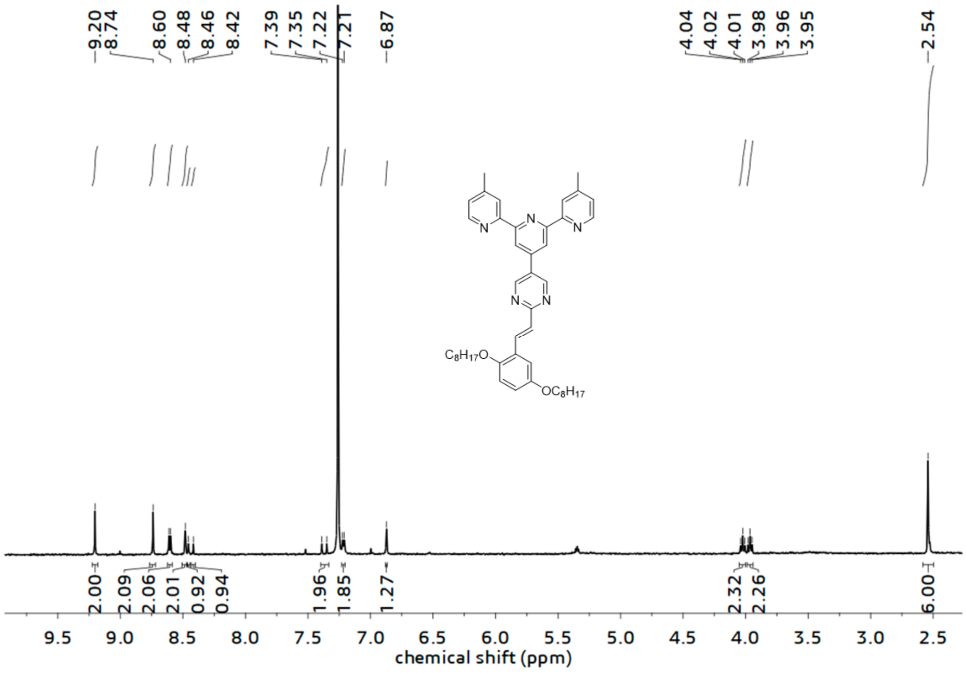

Figure S5. ^{1}H NMR spectrum of ligand **L3** in $CDCl_3$.

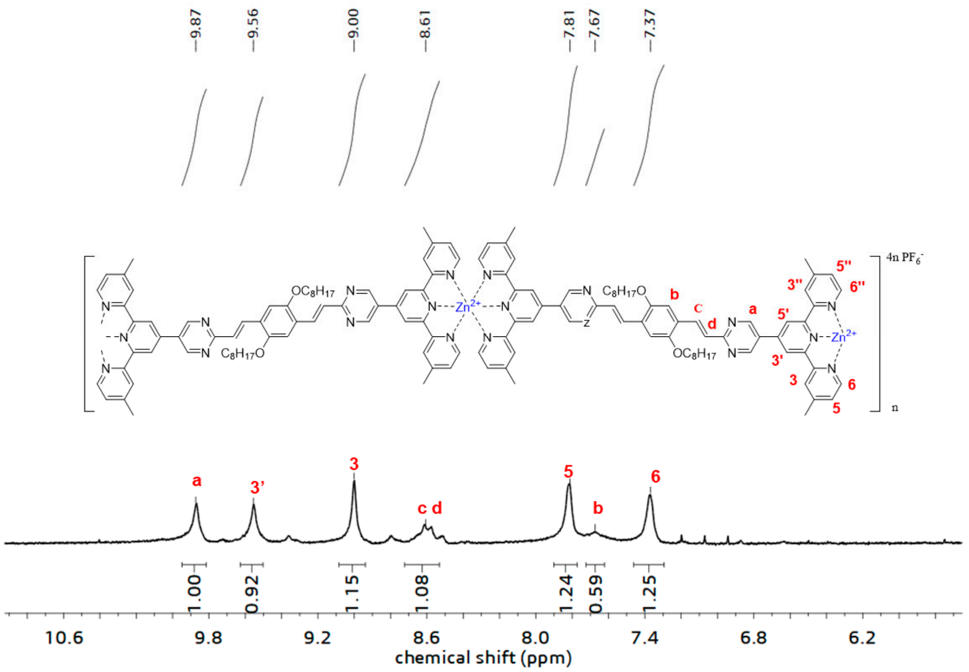

Figure S6. ^{1}H NMR spectrum of polymer **P2** in DMSO-d$_6$.

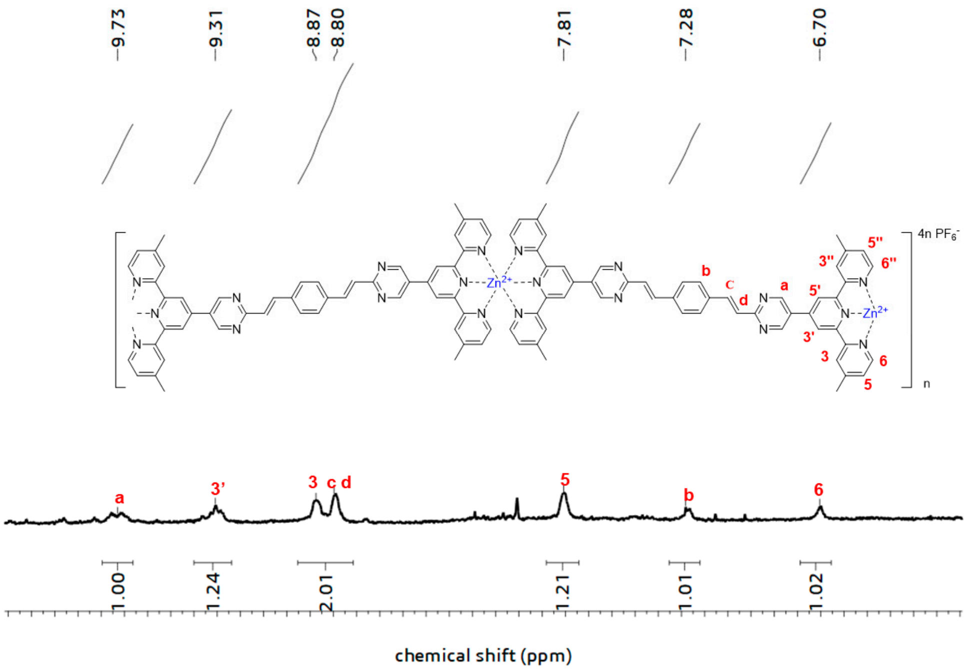

Figure S7. ^{1}H NMR spectrum of polymer **P1** in DMSO-d$_6$.

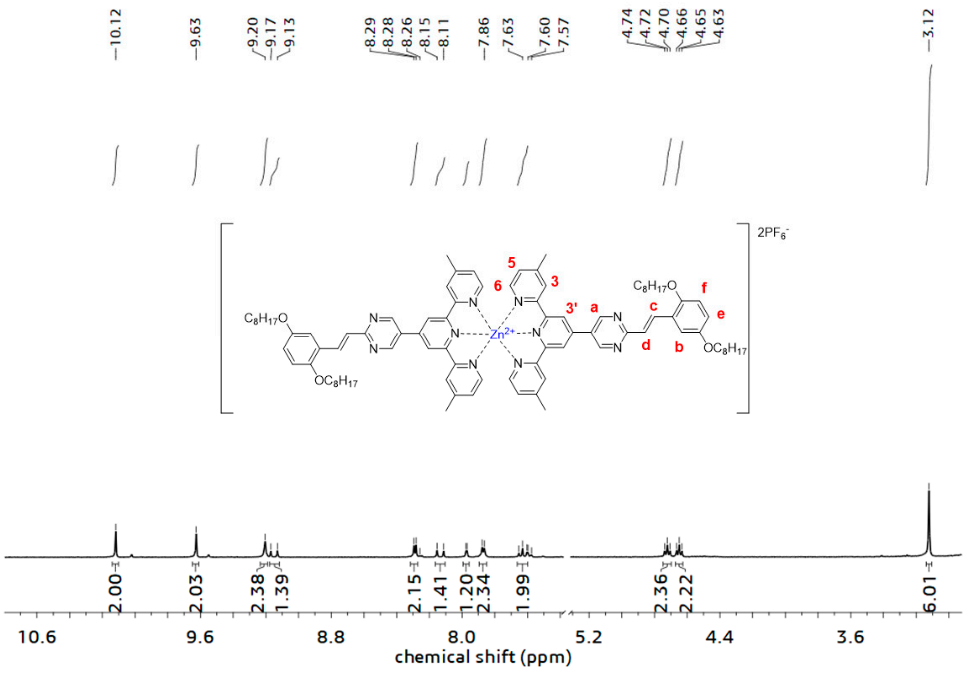

Figure S8. ^{1}H NMR spectrum of complex [**L3$_2$Zn**] in CD_3CN.

2D COSY NMR spectra

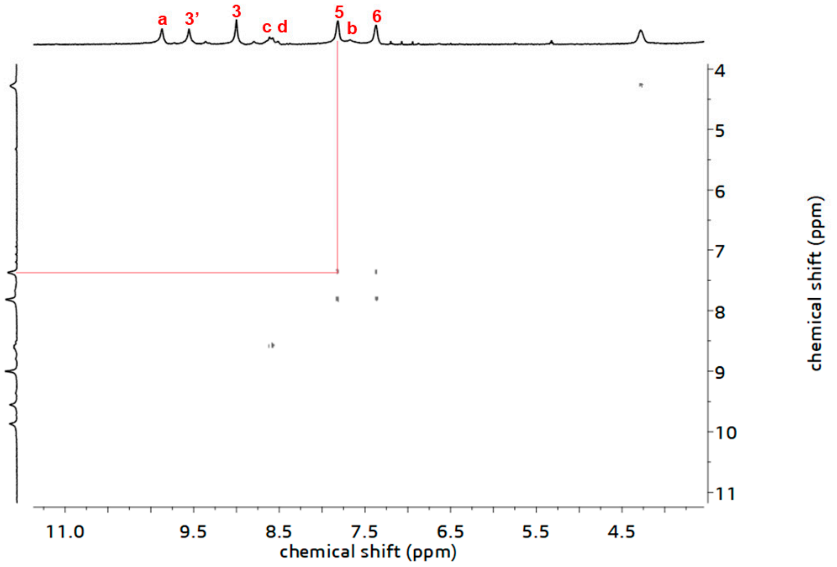

Figure S9. 2D COSY NMR spectrum of polymer **P2** in DMSO-d$_6$.

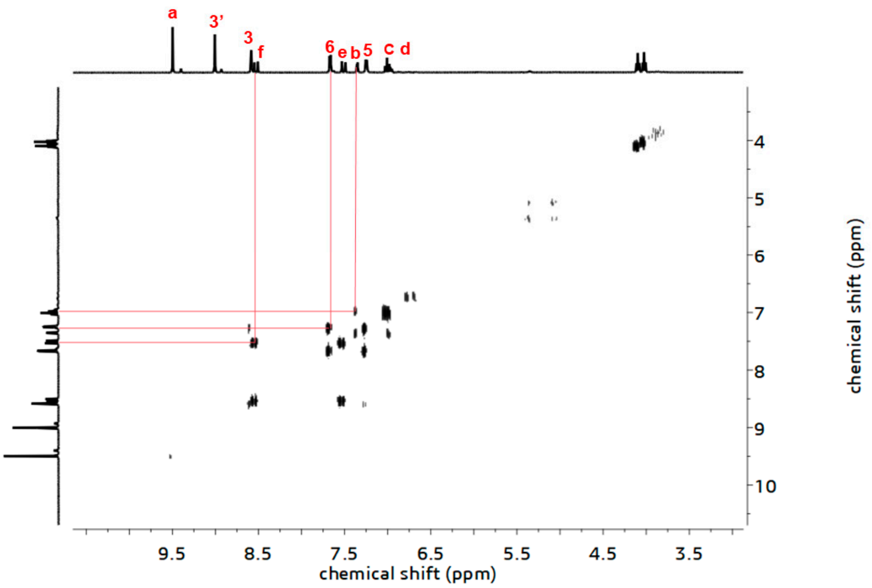

Figure S10. 2D COSY NMR spectrum of complex [**L3$_2$Zn**] in CD_3CN.

2D NOESY NMR spectra

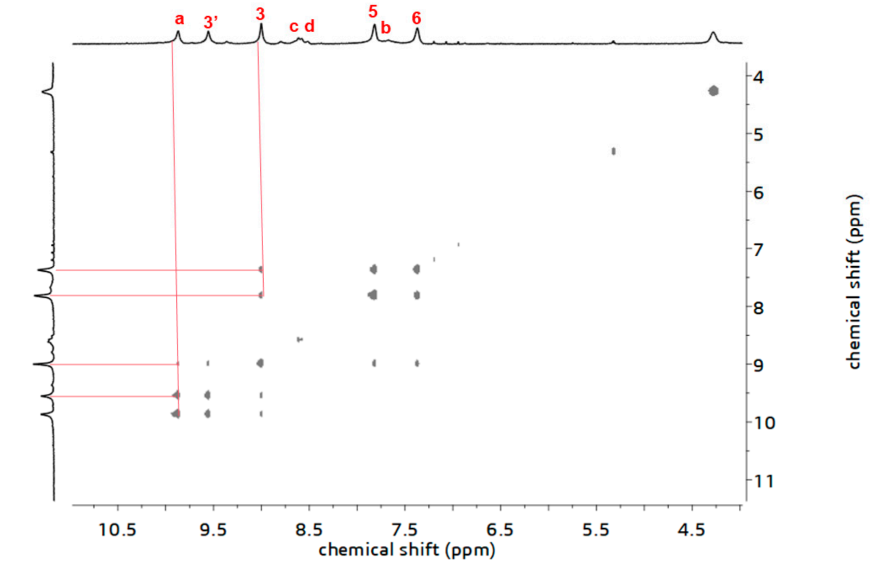

Figure S11. 2D NOESY NMR spectrum of **P2** in DMSO-d_6.

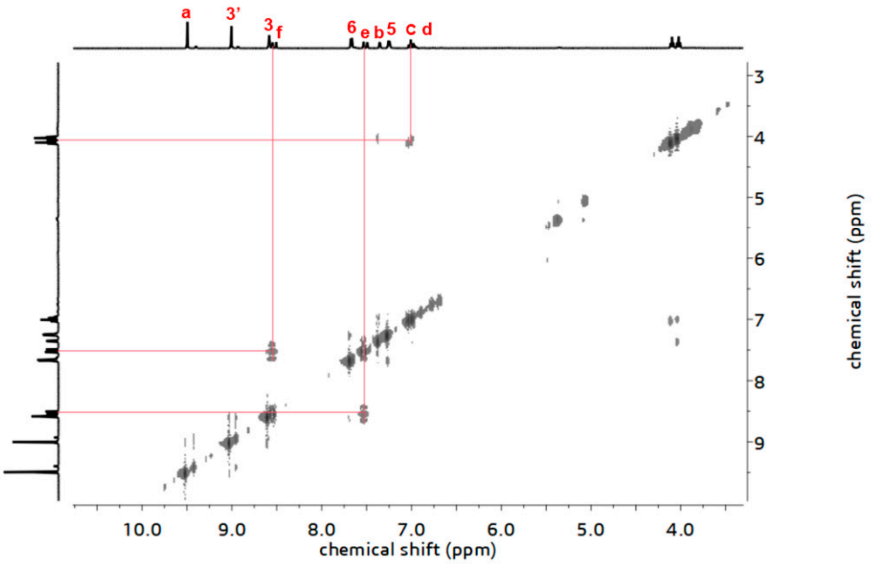

Figure S12. 2D NOESY NMR spectrum of complex [**L3$_2$Zn**] in CD_3CN.

^{13}C NMR spectra

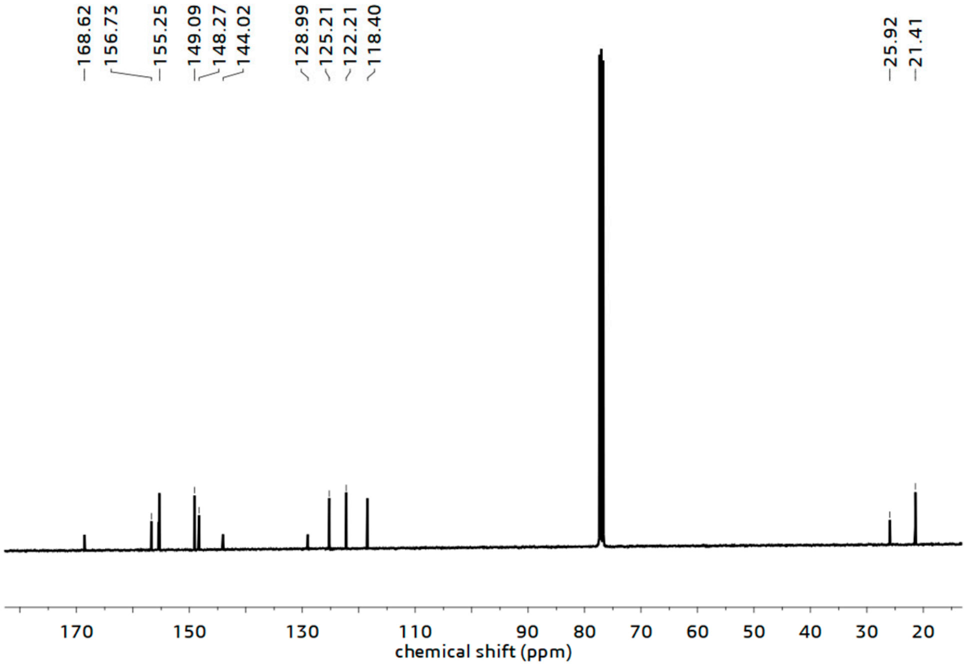

Figure S13. ^{13}C NMR spectrum of compound **1** in $CDCl_3$.

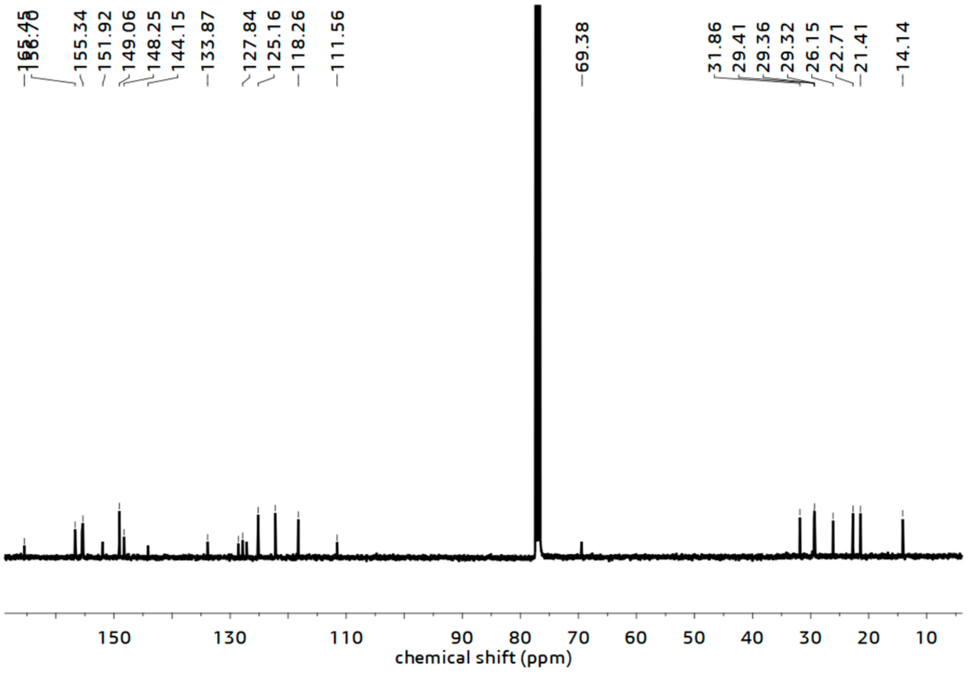

Figure S14. ^{13}C NMR spectrum of ligand **L2** in $CDCl_3$.

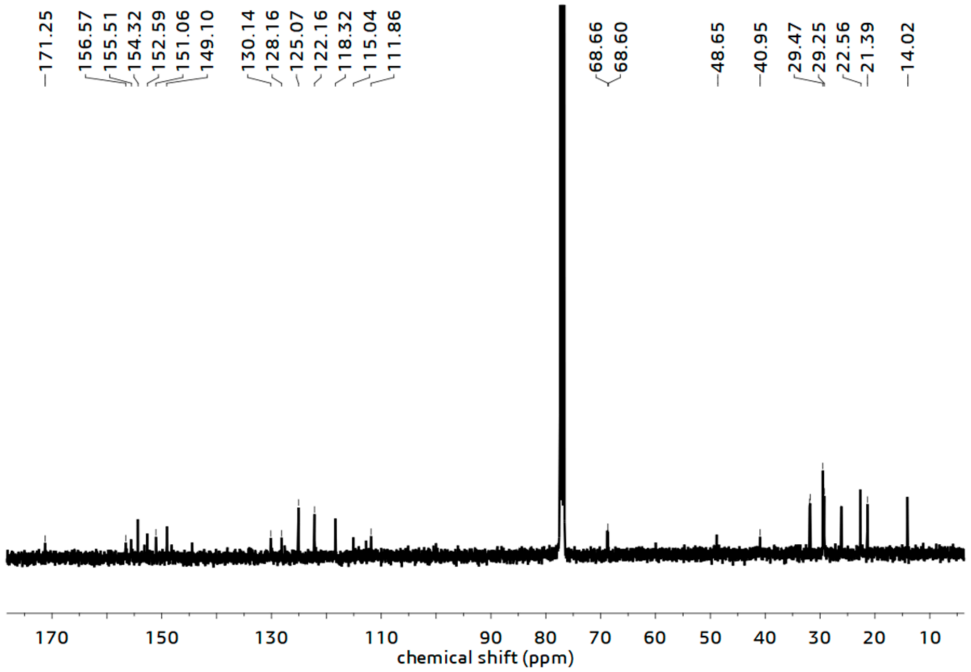

Figure S15. ^{13}C NMR spectrum of ligand **L3** in $CDCl_3$.

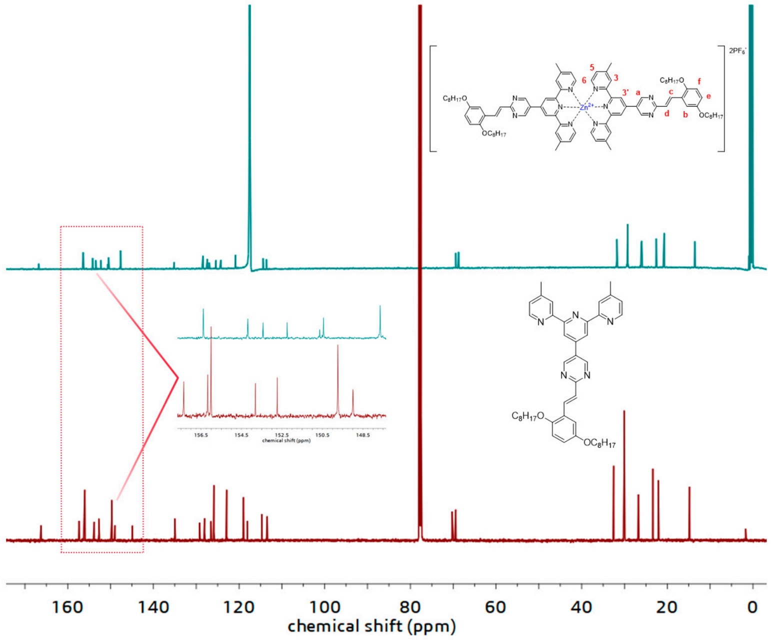

Figure S16. ^{13}C NMR spectra of **L3** and **L3$_2$Zn**.

S2 ESI-MS spectra

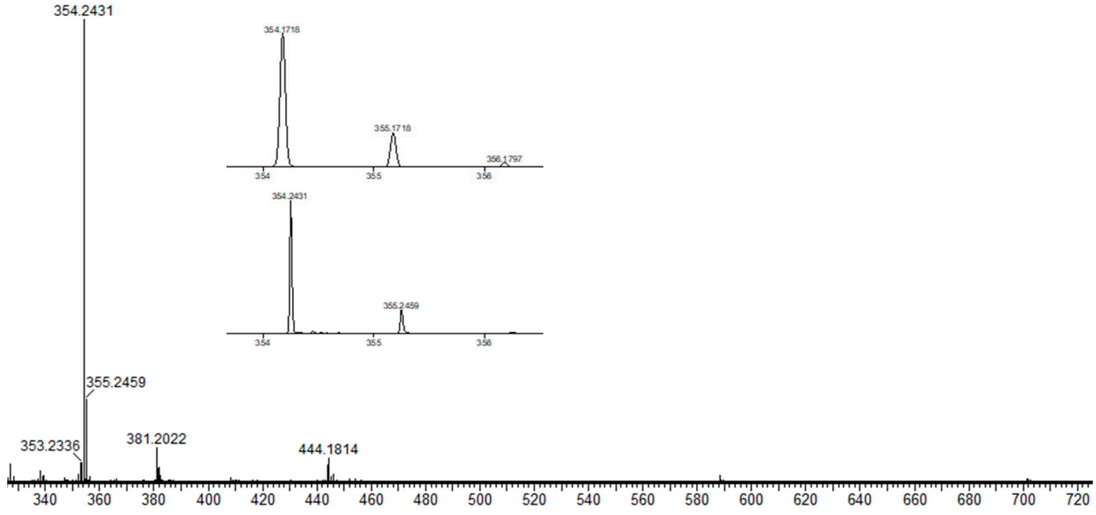

Figure S17. ESI-MS spectrum of compound **1**.

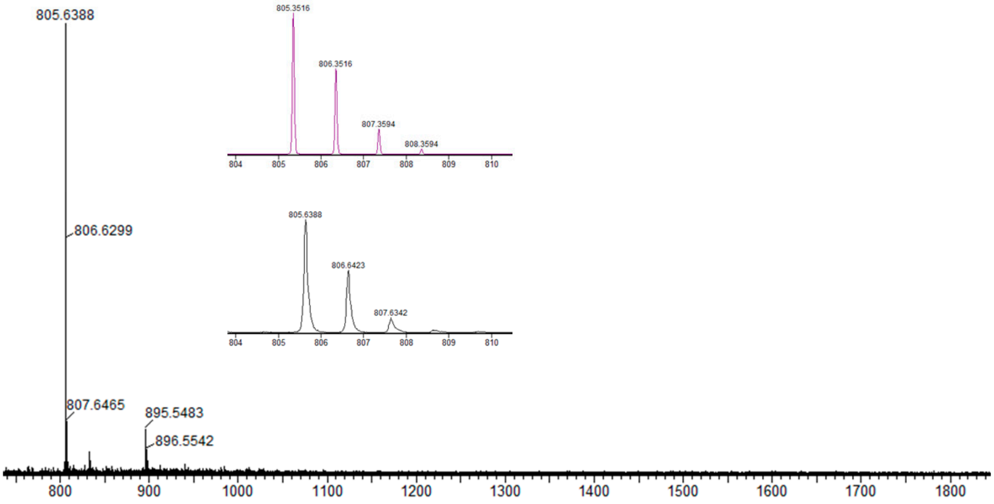

Figure S18. ESI-MS spectrum of ligand **L1**.

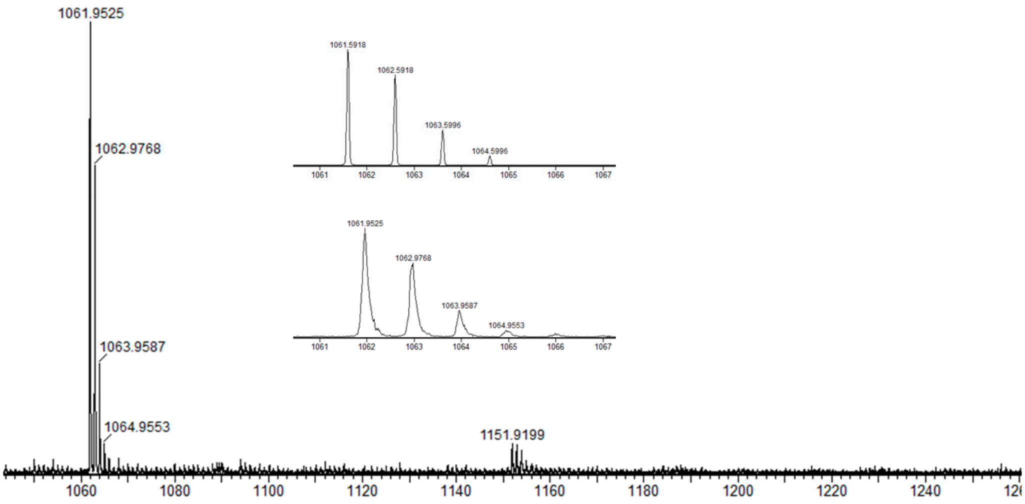

Figure S19. ESI-MS spectrum of ligand **L2**.

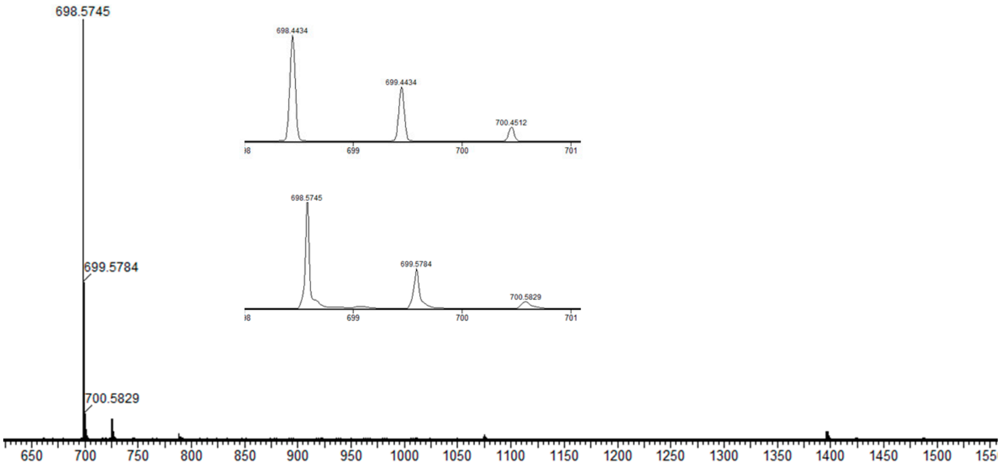

Figure S20. ESI-MS spectrum of ligand **L3**.

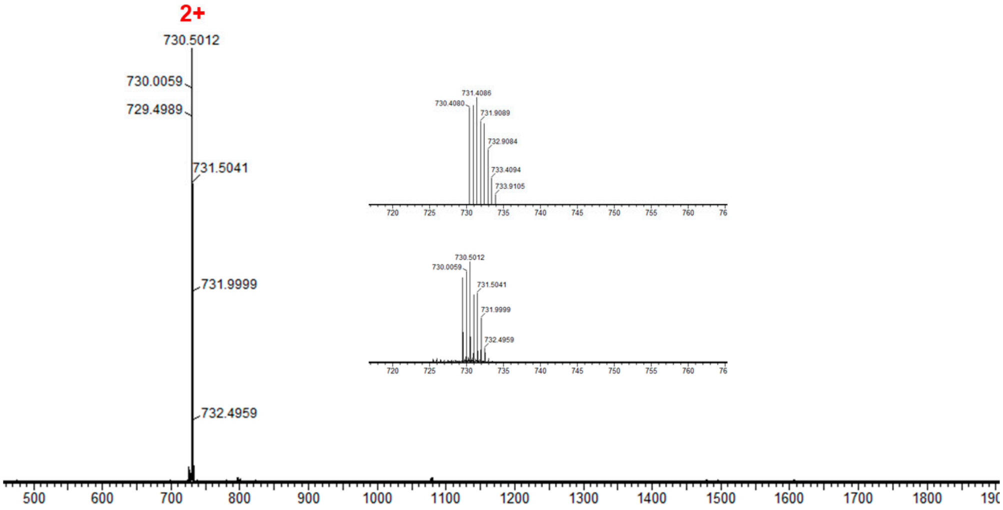

Figure S21. ESI-MS spectrum of complex [**L3**$_2$**Zn**].

S3 SEM and TEM images

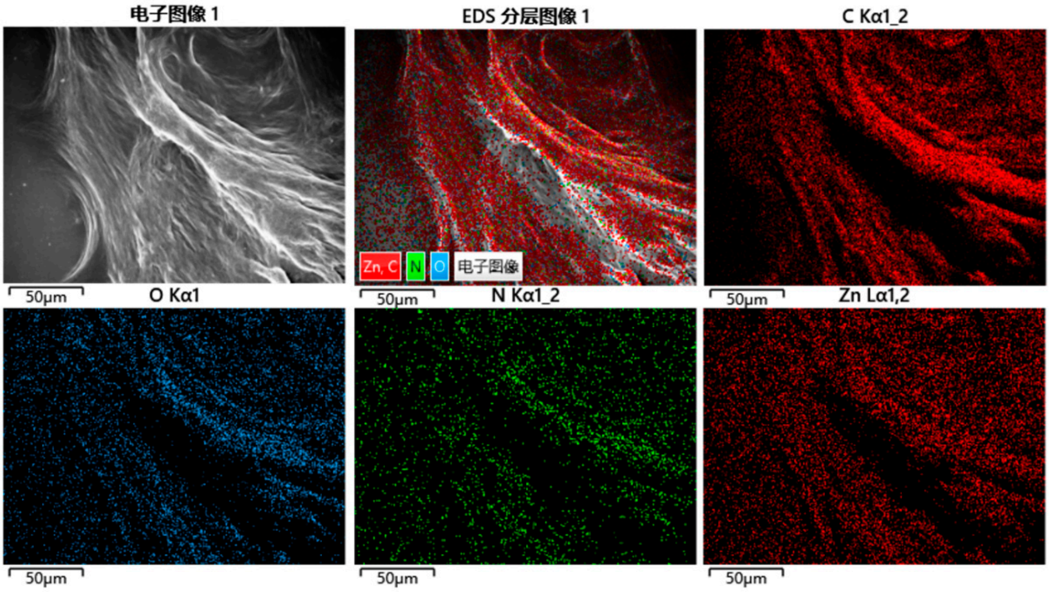

Figure S22. SEM and mapping images of solid **P2**.

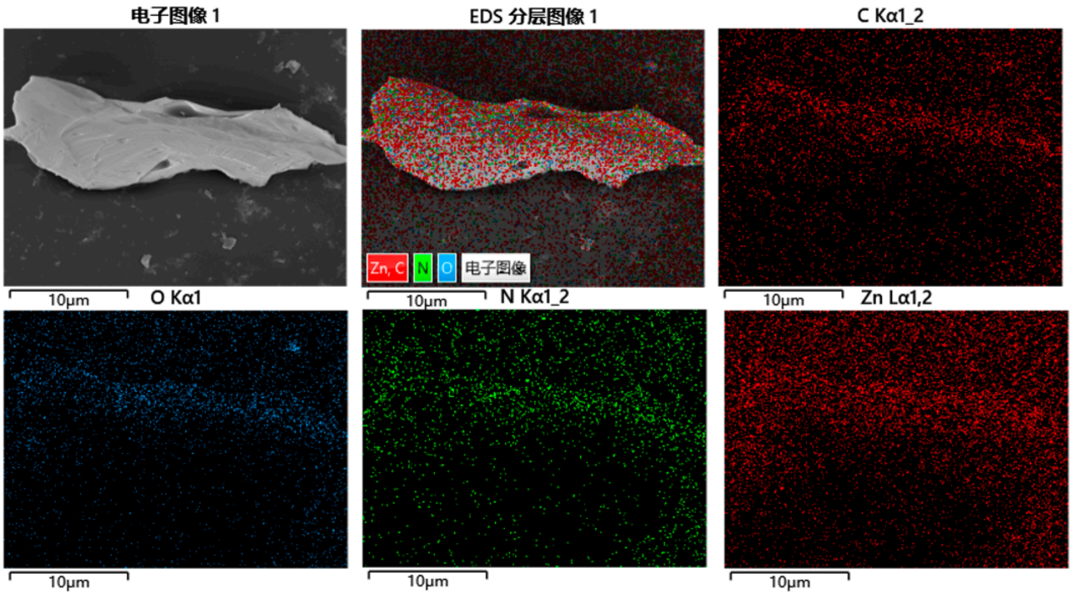

Figure S23. SEM and mapping images of solid **P1**.

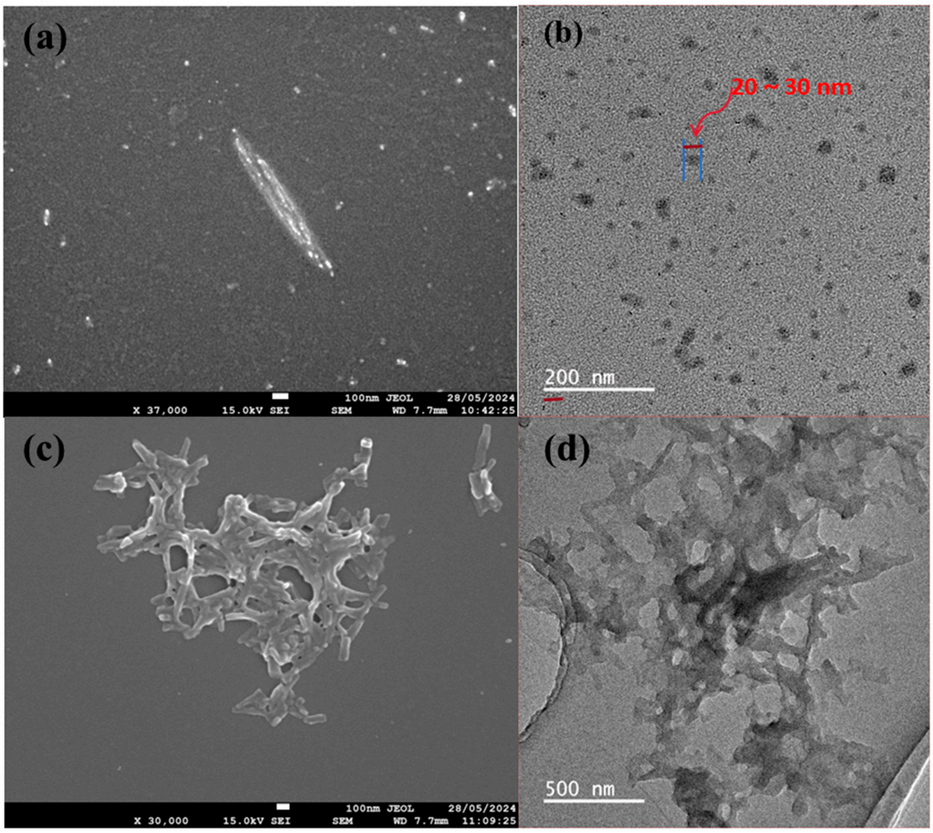

Figure S24. (a) SEM and (b) TEM images of **P2**; (c) SEM and (d) TEM images of **P1**.

S4 Optical property

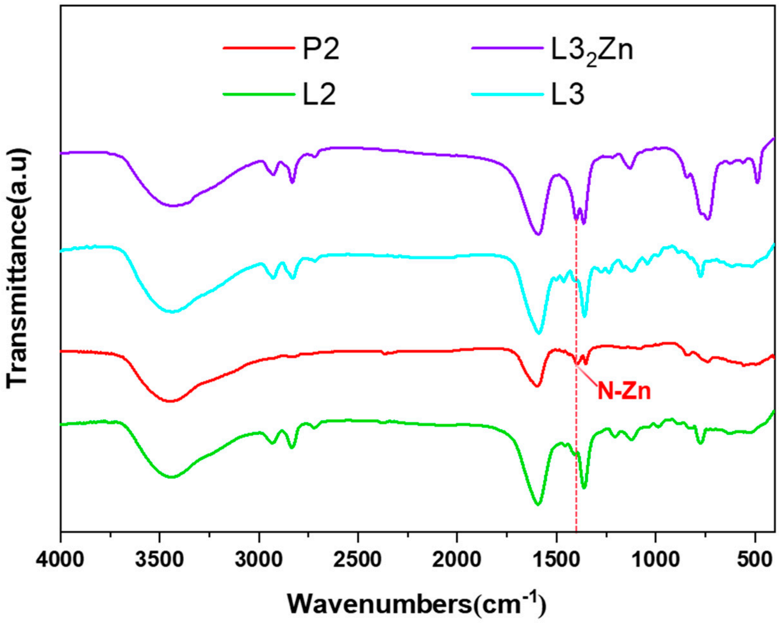

Figure S25. FT-IR spectrum of **L2**, **L3**, **P2** and **L3₂Zn**.

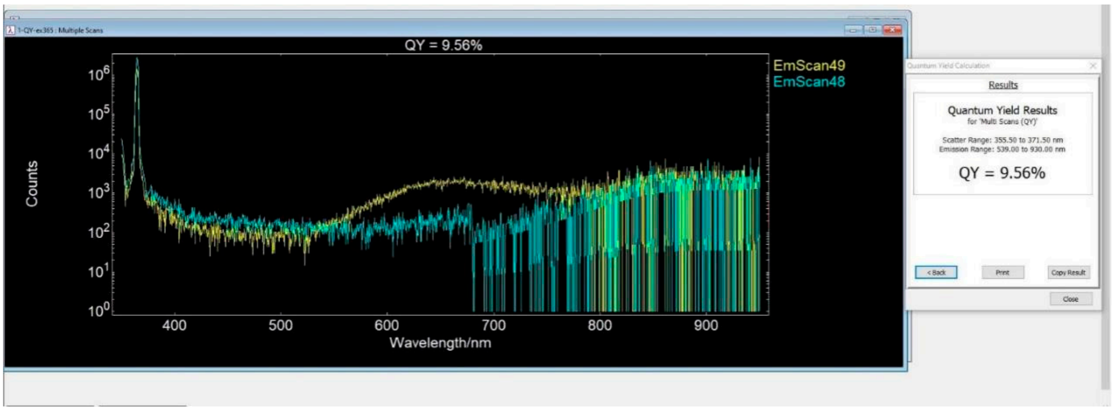

Figure S26. Fluorescence quantum yield of **P2**.

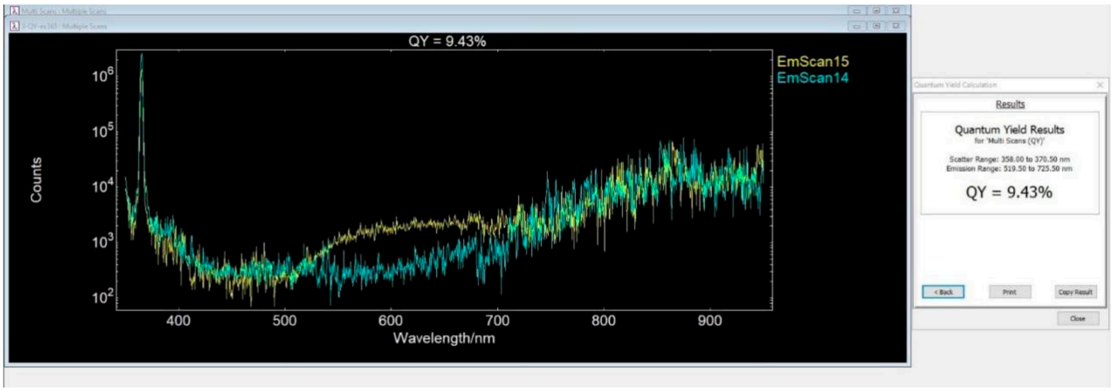

Figure S27. Fluorescence quantum yield of **P1**.

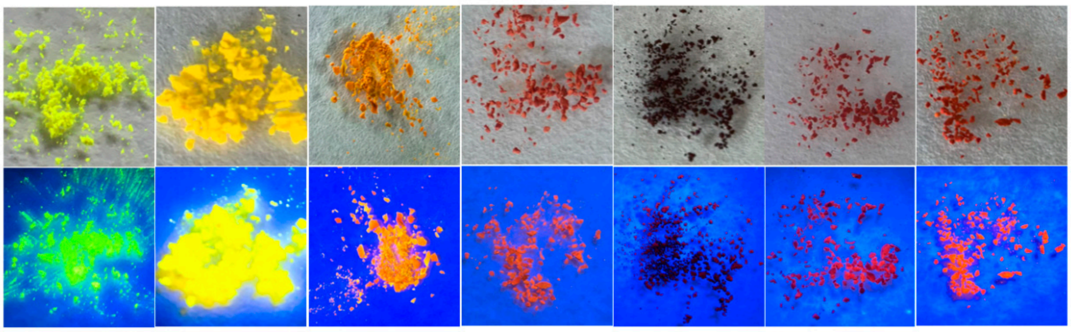

Figure S28. Photographs of **L1**, **L2**, **P1**, **P2**, **P2**-HCl, **P2**-HCl-7day, **P2**-HCl-NH_3H_2O under daylight and ultraviolet light.

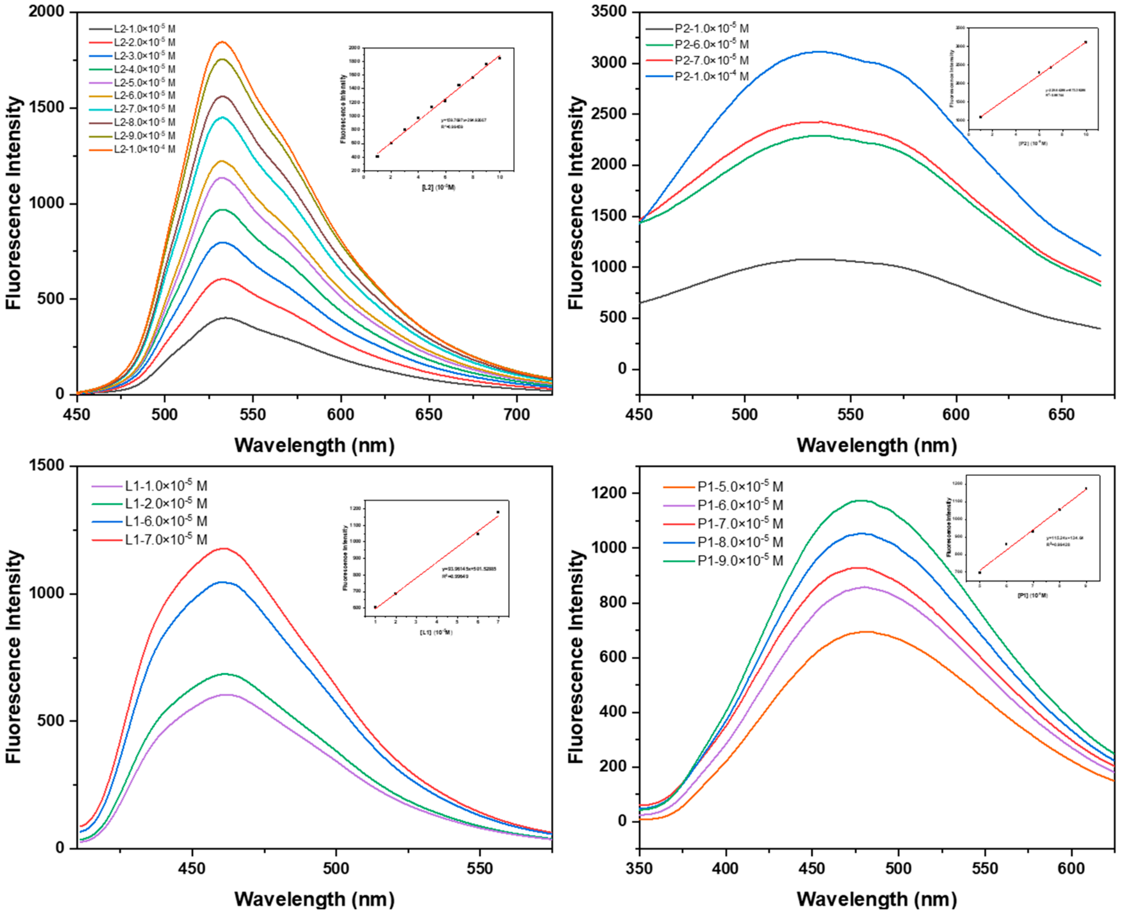

Figure S29. Fluorometric titration diagram of **L2**, **L1**, **P1**, **P2**. LOD(**L2**) =3δ/k =3×2.1317÷159.77×10^{-5}M=4.00×10^{-7} M, LOQ(**L2**)=10δ/k =1.22×10^{-6} M; LOD(**L1**) =3δ/k =6.8×10^{-7} M, LOQ(**L1**)=10δ/k =2.2×10^{-6} M; LOD(**P1**) =3δ/k =5.55×10^{-7} M, LOQ(**P1**)=10δ/k =1.85×10^{-6} M; LOD(**P2**) =3δ/k =2.8×10^{-7} M, LOQ(**P2**)=10δ/k =9.44×10^{-7} M.

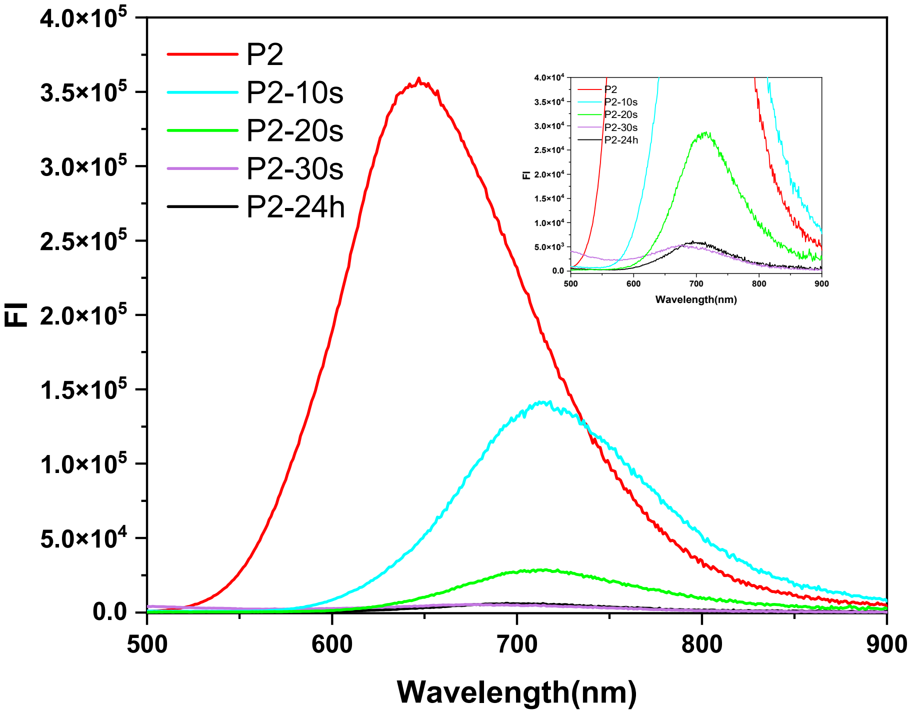

Figure S30. Time-dependent solid fluorescence spectra of polymer **P2** after protonation.

S5 Molecular model

Geometry optimization parameters	Energy parameters
Algorithm: Smart<br>Convergence tolerance:<br>Energy: 0.001 kcal/mol<br>Force: 0.5 kcal/mol/A<br>Maximum number of iterations: 5000<br>Motion groups rigid: NO	Forcefield: Universal<br>Charges: Use current<br>Electrostatic terms:<br>Summation method: Atom based<br>Truncation method: Cubic spline<br>Cutoff distance: 12.5 A<br>Spline width: 1 A<br>Buffer width: 0.5 A<br><br>van der Waals terms:<br>Summation method: Atom based

	Truncation method: Cubic spline<br>Cutoff distance: 12.5 A<br>Spline width: 1 A<br>Buffer width: 0.5 A

Table S1. The parameters of geometry optimization by using Forcite Calculation of Material Studio.

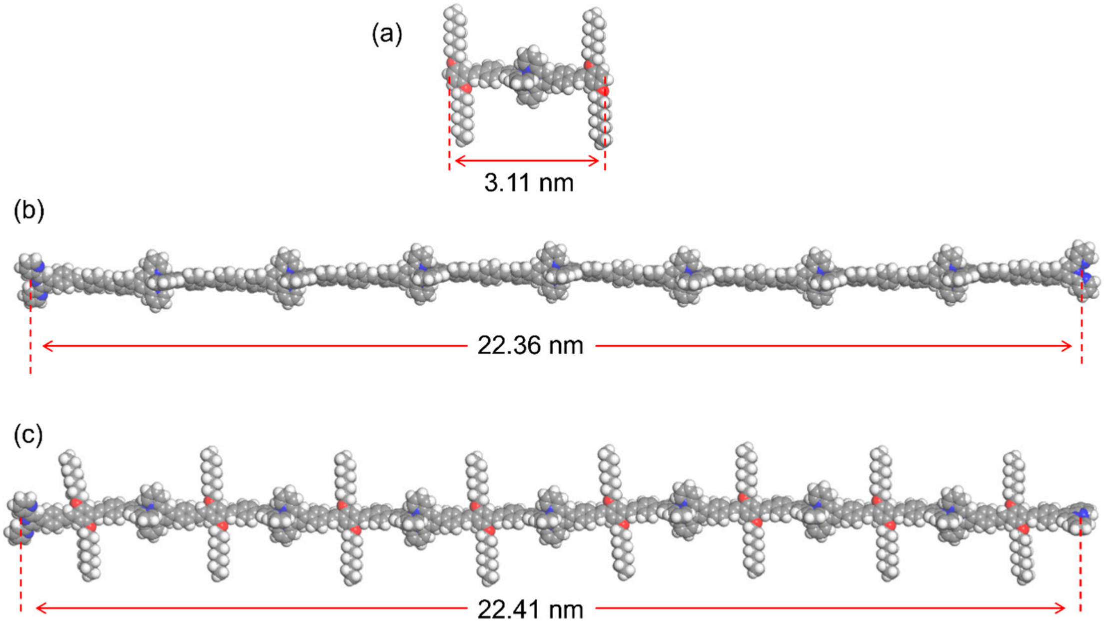

Figure S31. Geometry optimization molecule model and corresponding size of (a) complex [**L3**$_2$**Zn**], (b) polymer **P1** (DP = 8) and (c) polymer **P2** (DP = 8).

Reagent name	Purity	Provider
4-methyl-2-acetyl pyridine	98%	Bide Pharmatech Co.,Ltd
2-methyl-5-pyrimidine formaldehyde	97%	Bide Pharmatech Co.,Ltd
2,5-bis-(octyloxy)-terephthalaldehyde	98%	Bide Pharmatech Co.,Ltd
1-Bromooctane	99%	Bide Pharmatech Co.,Ltd
2,5-Dihydroxybenzaldehyde	98%	Bide Pharmatech Co.,Ltd
Sodium Hydroxide	98%	Greagent
Zinc nitrate hexahydrate	98%	Sinopharm Group Co. Ltd.

Ammonium hexafluorophosphate	98%	Bide Pharmatech Co.,Ltd
Methyl alcohol	99%	Adamas
Ethanol	99%	Adamas
Dichloromethane	99%	Greagent
Isohexane	AR	Greagent
Tetrahydrofuran	99%	Adamas
Bromotrichloromethane	99%	Sinopharm Group Co. Ltd.
Ammonium Hydroxide	25-28%	Sinopharm Group Co. Ltd.
Lsopropyl Alcohol	99%	Sinopharm Group Co. Ltd.

Table S2. The provider and purity of chemical reagents.
